# An update of the Worldwide Integrated Assessment (WIA) on systemic pesticides. Part 4: Alternatives in major cropping systems

**DOI:** 10.1007/s11356-020-09279-x

**Published:** 2020-06-04

**Authors:** Andrea Veres, Kris A. G. Wyckhuys, Jozsef Kiss, Ferenc Tóth, Giovanni Burgio, Xavier Pons, Carlos Avilla, Stefan Vidal, Jaka Razinger, Renata Bazok, Ewa Matyjaszczyk, Ivan Milosavljević, Xuan Vi Le, Wenwu Zhou, Zeng-Rong Zhu, Hagus Tarno, Buyung Hadi, Jonathan Lundgren, Jean-Marc Bonmatin, Maarten Bijleveld van Lexmond, Alexandre Aebi, Aunu Rauf, Lorenzo Furlan

**Affiliations:** 1 Department of Integrated Plant Protection / Plant Protection Institute, Szent István University (SZIE), Páter Károly út 1, Gödöllő, 2103 Hungary; 2 China Academy of Agricultural Sciences, Beijing, China; 3 University of Queensland, Brisbane, Australia; 4 Fujian Agriculture and Forestry University, Fuzhou, China; 5 Chrysalis, Hanoi, Vietnam; 6 Department of Agricultural and Food Sciences, Alma Mater Studiorum University of Bologna (UNIBO), Bologna, Italy; 7 University of Lleida, Lleida, Spain; 8 University of Seville, Seville, Spain; 9 Department of Crop Sciences/Agricultural Entomology, Georg-August-University, Göttingen, Germany; 10 Agricultural Institute of Slovenia, Ljubljana, Slovenia; 11 Department for Agricultural Zoology, University of Zagreb Faculty of Agriculture, Zagreb, Croatia; 12 Institute of Plant Protection – National Research Institute, Poznań, Poland; 13 Department of Entomology, University of California, Riverside, CA USA; 14 Plant Protection Research Institute, Hanoi, Vietnam; 15 Institute of Insect Sciences, Zhejiang University, Hangzhou, China; 16 Brawijaya University, Malang, Indonesia; 17 IRRI Cambodia Office, Phnom Penh, Cambodia; 18 Ecdysis Foundation, Estelline, USA; 19 Centre de Biophysique Moléculaire, Centre National de la Recherche Scientifique (CNRS), Orléans, France; 20 IUCN, Neuchâtel, Switzerland; 21 Laboratory of Soil Biodiversity, Institute of Ethnology, University of Neuchâtel, Neuchâtel, Switzerland; 22 Bogor Agricultural University, Bogor, Indonesia; 23 Agricultural Research Department, Veneto Agricoltura, Legnaro, Italy

**Keywords:** Neonicotinoids, Soil pests, Aphids, Whitefly, Brown planthopper, Agricultural policy, IPM, Biological control

## Abstract

We present a synthetic review and expert consultation that assesses the actual risks posed by arthropod pests in four major crops, identifies targets for integrated pest management (IPM) in terms of cultivated land needing pest control and gauges the implementation “readiness” of non-chemical alternatives. Our assessment focuses on the world’s primary target pests for neonicotinoid-based management: western corn rootworm (WCR, *Diabrotica virgifera virgifera*) in maize; wireworms (*Agriotes* spp.) in maize and winter wheat; bird cherry-oat aphid (*Rhopalosiphum padi*) in winter wheat; brown planthopper (BPH, *Nilaparvata lugens*) in rice; cotton aphid (*Aphis gossypii*) and silver-leaf whitefly (SLW, *Bemisia tabaci*) in cotton. First, we queried scientific literature databases and consulted experts from different countries in Europe, North America, and Asia about available IPM tools for each crop-pest system. Next, using an online survey, we quantitatively assessed the economic relevance of target pests by compiling country-level records of crop damage, yield impacts, extent of insecticide usage, and “readiness” status of various pest management alternatives (i.e., research, plot-scale validation, grower-uptake). Biological control received considerable scientific attention, while agronomic strategies (e.g., crop rotation), insurance schemes, decision support systems (DSS), and innovative pesticide application modes were listed as key alternatives. Our study identifies opportunities to advance applied research, IPM technology validation, and grower education to halt or drastically reduce our over-reliance on systemic insecticides globally.

**Electronic supplementary material:**

The online version of this article (10.1007/s11356-020-09279-x) contains supplementary material, which is available to authorized users.

## Introduction

Neonicotinoid insecticides are extensively used against multiple herbivorous insects in annual crops across the globe (Furlan and Kreutzweiser 2015; Simon-Delso et al. 2015). Their in-field application is primarily preventative and unguided, thus conflicting with globally accepted principles of integrated pest management (IPM) (Furlan et al. 2017a; Kogan 1998; Pedigo and Rice 2014; Barzman et al. 2015). Scant information is available on the actual benefits of these products in terms of crop performance, economic yield or farm-level profit, and the few existing peer-reviewed studies demonstrate how yield benefits of, e.g., neonicotinoid seed coatings are routinely negligible (Bredeson and Lundgren 2015b; Furlan and Kreutzweiser 2015; Bredeson and Lundgren 2015a; Matyjaszczyk et al. 2015; Milosavljević et al. 2019; Furlan et al. 2018). Globally, efforts are being made to reduce farmer reliance on and overuse of chemical pesticides—including applied research, farm-level validation and communication of alternative pest management options (Horgan 2017). For example, in the European Union (EU), Commission Directive 2009/128/EC enables vigilance over the sustainable use of chemically synthesized pesticides, secures a continuing reliance upon IPM, and provides the legal framework to pursue a lasting, far-reaching reduction in the prophylactic usage of systemic insecticides (Furlan et al. 2018). Despite this increased attention, global insecticide use has increased dramatically in recent decades and farmers in multiple countries now favor prophylactic pest management approaches over IPM (Horgan 2017).

Worldwide, neonicotinoids are used to control a wide range of herbivorous insects in broad-acre crops, such as maize (globally cultivated on 171 M ha), wheat (220 M ha), rice (paddy; 162 M ha) and cotton (approximately 33 M ha). As the world’s most widely used insecticides, neonicotinoids currently represent 25% of the global insecticide market (Hladik et al. 2018). One of the key concerns regarding neonicotinoids is their routine, prophylactic use as “convenience” pesticides, i.e., seed coatings, stem dips or drench applications (Mourtzinis et al. 2019). While seed coatings indeed lessen the amount of overspray and drift, the products readily leach into the soil and water phase and are widely detected in the broader farming environment (i.e., surface waters, plant pollen, nectar, and other exudates) (Bonmatin et al. 2015; Alford and Krupke 2019). Furthermore, the consumption of active substance per hectare is several times higher for seed treatment than for (targeted) foliar applications (Matyjaszczyk 2017). Lastly, their “blanket” application at the time of planting (or crop seeding) interferes with the action of natural enemies and thus compromises natural, cost-free biological control (Seagraves and Lundgren 2012; Douglas and Tooker 2016). As such, neonicotinoid insecticides contribute to biodiversity loss, impact environmental health, and undermine ecological resilience of farmland ecosystems (Eng et al. 2019; Humann-Guilleminot et al. 2019a; Sánchez-Bayo and Wyckhuys 2019).

Farmers’ dependency on neonicotinoid insecticides is of global concern and has continued unabated in the United States (US) for over two decades (Jeschke et al. 2011; Simon-Delso et al. 2015; Douglas and Tooker 2015; Hladik et al. 2018). Following the first registration of imidacloprid in 1991, six other active ingredients were released on the US market (Bass et al. 2015). In the mid-2000s, active marketing of insecticide-coated seeds, mounting insecticide resistance issues, and public concern over high mammalian toxicity of older products led to a surge in neonicotinoid usage over extensive geographical areas, e.g., US Midwest (Meehan and Gratton 2016). By 2014, 1.16 million kg/year of thiametoxam was applied to US corn, soybean, wheat, and cotton (NAWQA 2014). At present, neonicotinoid seed coatings are used in virtually 100% of the conventional maize planted in the US and canola in Canada. Seed coatings are equally used in other crops (i.e., soybean, oilseed rape, cereals, rice, cotton, sunflower) in the USA and across the globe (Hladik et al. 2018; Douglas and Tooker 2015; Esser et al. 2015).

In the EU, the first neonicotinoid (imidacloprid) was registered in the mid 1990s. Few years after neonicotinoids approval, bee health problems and other non-target impacts have surfaced (Pistorius et al. 2009, Doucet-Personeni et al. 2003; Henry et al. 2012; Whitehorn et al. 2012; Alix and Mercier 2009; Bortolotti et al. 2009; Burgio et al. 2012). Subsequently, in some EU member states, the use of particular active ingredients was suspended on certain crops in pollinator habitats, e.g., on sunflower and oilseed rape in France (Maxim and van der Sluijs 2013), or on maize in Italy (Bortolotti et al. 2009). In Italy, after this precautionary suspension, the number of bee mortality events linked to maize cultivation drastically declined while maize yield levels remained unchanged (Sgolastra et al. 2017). Yet, neonicotinoids were deemed to be “essential” components to EU farming, and their continent-wide suspension was anticipated to cause unacceptable production losses and (socio-)economic upheaval (Noleppa and Hahn 2013). The ensuing debate juxtaposed these presumed (socio-)economic impacts against an increase in primary productivity and farm profitability related to reconstituted pollination and other biodiversity-based ecosystem services (Garibaldi et al. 2014), while scientific information did not report any benefits of neonicotinoids on crop yields (Seltenrich 2017), quality of harvested produce, or farm-level profitability (LaCanne and Lundgren 2018). Parallel to the accumulating scientific evidence of their deleterious effects on human health (Seltenrich 2017) and biodiversity, including vertebrate and invertebrate wildlife (Chagnon et al. 2015; Pisa et al. 2015; Gibbons et al. 2015; Humann-Guilleminot et al. 2019b; Pisa et al. 2017; Sánchez-Bayo et al. 2016; Lundin et al. 2015; Tosi et al. 2017; Taliansky-Chamudis et al. 2017; Pecenka and Lundgren 2015; Douglas et al. 2015), there was a disclosure of scientific arguments and field observations (Blacquière et al. 2012; Carreck and Ratnieks 2014; Cresswell et al. 2012). From 2012 onward, the European Food Safety Authority (EFSA) suggested to suspend certain uses of active ingredients, and by March 2017, the European Commission had proposed a ban of all outdoor usage of three neonicotinoid pesticides (i.e., imidacloprid, clothianidin, and thiamethoxam). However, crops that are considered “non-attractive” to honeybees, e.g., cereals, sugar beet, occupy the largest surface of arable land in Europe (approx. 60%, Eurostat 2017), still receive considerable rates of neonicotinoid application (Simon-Delso et al. 2015), and current application schemes equally pose risk to non-target organisms, including natural enemies and pollinators (Calvo-Agudo et al. 2019). Certainly, as neonicotinoids affect all ecosystem components, a sole focus on (domesticated) honeybees is insufficient (Pisa et al. 2017).

Multiple efforts have also been made in North-America to counteract the above trends: the United States Environmental Protection Agency (USEPA) recently re-evaluated the registration of neonicotinoids, especially in relation to pollinators (USEPA 2014); the Government of Ontario (Canada) pursued an 80% cut in the use of neonicotinoid-treated maize and soybean seed by 2017 (Gov. of Ontario 2015). In 2011, imidacloprid was voluntarily withdrawn from use on bee-pollinated almonds in California, while other products are on the Pesticide Action Network (PAN) International’s list of highly hazardous pesticides (2010) for global phase-out, because of their toxicity to bees or potential role as carcinogens (i.e., thiacloprid). In China, one of the world’s key suppliers of neonicotinoid insecticides, domestic use is considerable, especially in the country’s 30 million hectare rice crop (Shao et al. 2013). Presently, six types of neonicotinoid are registered for domestic use. Whereas imidacloprid, acetamiprid, nitenpyram, and thiacloprid are developed by companies outside China, paichongding and imidaclothiz are newly developed compounds with independent intellectual property rights.

In maize (*Zea mays* (L.)), two key pests are targeted with neonicotinoids: (1) the western corn rootworm (WCR), *Diabrotica virgifera* ssp. *virgifera* LeConte (Coleoptera: Chrysomelidae) and (2) a complex of wireworm species (*Agriotes* spp. Coleoptera: Elateridae). For *D. virgifera* control, farmers typically apply granular soil insecticides or insecticide-coated seeds against the larvae and foliar insecticides against the adults (Ward et al. 2004). The combined cost of soil insecticides and aerial sprays, plus the yearly pest-inflicted crop losses, approached US$1 billion annually in the 1980s (Metcalf 1986). Following its 1990s detection and spread in Europe, the damage potential of *D. virgifera*—in the absence of control—was estimated at 472 million Euro annually (Wesseler and Fall 2010). Wireworms (*Agriotes brevis* Candeze*, A. sordidus* Illiger, and *A. ustulatus* Schäller) are of primary concern in Central and Southern Europe, with underground larval feeding resulting in root damage in maize and other cereal crops (Furlan 1996, 2004; Furlan 2014; Furlan and Tóth 2007). At present, soil insecticides are the preferred mode of wireworm control, impeding a further deployment of IPM strategies (Furlan 2005; Furlan and Kreutzweiser 2015). The bird cherry-oat aphid *Rhopalosiphum padi* (Linnaeus) causes direct feeding damage on cereals, transmits plant pathogens (e.g., barley yellow dwarf virus) and interferes with photosynthesis through its extensive production of honeydew (Dedryver et al. 2010; Mann et al. 1997; Finlay and Luck 2011; Harrington et al. 2007). Since the late 1980s, *R. padi* control has primarily been based on seed treatments and insecticide sprays, raising environmental concerns (Bredeson et al. 2015; Dedryver et al. 2010). In rice, the brown planthopper (BPH) *Nilaparvata lugens* (Stål) can cause up to 70% yield losses through direct feeding and virus transmission—with aggregate economic losses estimated at $20–100 million/year for India, Indonesia, Philippines, Japan and Taiwan (Heinrichs and Mochida 1984; Bateman 2016). Considered a “Green Revolution” pest, rice planthoppers first gained prominence in tropical Asia during the 1960s and 1970s when high-yielding rice varieties were actively promoted together with synthetic pesticides (Bottrell and Schoenly 2012; Escalada and Heong 2004). Brown planthopper outbreaks are due to the insecticide-induced mortality of natural enemies and an ensuing loss of regulating ecosystem services (Horgan and Crisol 2013; Bottrell and Schoenly 2012; Horgan 2018; Spangenberg et al. 2015) plus the widespread development of brown planthopper resistance to insecticides, including neonicotinids (Zhang et al. 2016; Min et al. 2014; Basanth et al. 2013; Puinean et al. 2010; Hadi et al. 2015; Matsumura et al. 2008) and insecticide-induced hormesis in which insecticide treatments enhance brown planthopper survival, development and reproduction (Yin et al. 2014; Nanthakumar et al. 2012; Horgan 2018; Zhu et al. 2004; Azzam et al. 2009; Azzam et al. 2011). Though brown planthopper-resistant rice varieties have been developed, widespread adaptation to resistance genes has compromised their effectiveness and insecticide applications thus remain the mainstay of Asian rice farmers (Spangenberg et al. 2015; Horgan 2018). In cotton, the cotton aphid, *Aphis gossypii* Glover and the silverleaf whitefly, *Bemisia tabaci* Gennadius inflict crop damage through direct feeding, virus vectoring and the extensive secretion of honeydew (Lu et al. 2012; Dedryver et al. 2010). The heightened adoption of *Bacillus thuringiensis* (Bt) transgenic cotton—for lepidopteran pest control—has led to increases in sap-feeder populations such as *B. tabaci* or *A. gossypii* (Lu et al. 2012). Though genetically engineered crops are well-suited to support biological control (Romeis et al. 2018), the increased infestation pressure of these sap-feeders has instead triggered the use of systemic insecticides (Deguine et al. 2008). Furthermore, rapidly evolved resistance to neonicotinoids has been recorded, e.g., in Australia (Herron and Wilson 2011), West Africa (Houndété et al. 2010), and Asia (Koo et al. 2014; Matsuura and Nakamura 2014; Ahmad and Arif 2008) with a resulting intensification of pesticide use and accompanying harmful impacts on resident natural enemy communities (Gerling and Naranjo 1998; Naveed et al. 2008; Yao et al. 2015; Sohrabi et al. 2012).

Aside from neonicotinoid insecticides, numerous management techniques have been investigated for the above crop × pest complexes; many with highly favorable environmental, human health, and socio-economic profiles. Nature-based innovations (e.g., biological control) can be deployed in open-field and protected cultivation, have proven advantages over insecticides (Bommarco et al. 2011; Naranjo et al. 2015; Shields et al. 2019) and favorable food safety profiles (Bale et al. 2008), yet are faced with globally lagging rates of grower adoption and deficient stakeholder awareness (Shields et al. 2019; van Lenteren 2012; Wyckhuys et al. 2018; Wyckhuys et al. 2019c; Barratt et al. 2018). Other approaches such as pest insurance schemes (Quiggin et al. 1993; Miranda and Vedenov 2001), ecological engineering (e.g., (Gurr et al. 2016), pesticide taxes coupled with enhanced grower education (Praneetvatakul et al. 2013), regenerative farming (LaCanne and Lundgren 2018), customized decision-support tools, cultural control, and other IPM options have equally been examined. Many of these practices constitute part of an agricultural systems “redesign”—a necessary component for transformative change in the world’s agriculture sector, and a core component of sustainable intensification (Pretty et al. 2018). For certain technologies, considerable progress has been made on the research front, yet advances in (on-farm, participatory) technology validation, farmer extension or wide-ranging diffusion have been limited. Some technologies have been readily adopted, validated, and adapted by individual growers or (small- to mid-size) farmer nuclei in certain countries, and these successes now wait to be communicated, up-scaled and transferred to other areas (Westphal et al. 2015; Gurr et al. 2016).

The present paper is the continuation of the Worldwide Integrated Assessment (WIA) on systemic insecticides published in 2015 which included alternative aspects (Furlan and Kreutzweiser 2015), and then of the WIA update published in 2018 which also included a chapter on alternatives (Furlan et al. 2018). Here, we provide a systematic assessment of alternatives to neonicotinoids for the management of key arthropod pests in four arable crops of global relevance (i.e., maize, cotton, rice, and winter wheat). More specifically, we draw upon (i) a review of scientific literature, (ii) an expert consultation involving 16 scientists and crop protection professionals from multiple countries on a crop-specific and geographically explicit “readiness” assessment of pest management alternatives, and (iii) a risk assessment of pest outbreaks, as conducted through online survey tools. Our systematic assessment of the state of development (i.e., research, plot-scale validation, grower-uptake) for a select set of technologies constitutes a valuable resource for scientists, pest management professionals, extension officers, agro-enterprises, and individual farmers. Our work also informs policy dialogue and can create global traction for the “redesign” of farming systems founded upon agro-ecology, ecologically centered IPM, and arthropod biological control.

## Materials and methods

### Literature review

Two scientific literature databases (i.e., Springerlink and Sciencedirect) were queried using keyword combinations specific to the different crop × pest systems: “*Diabrotica* AND control AND maize” and “*Agriotes* AND control AND maize,” etc. Searches were limited to articles published in English from January 1999 until March 2017. All publication abstracts were screened, and literature references were selected based on their relevance for crop protection in the seven target crop × pest systems, i.e., *Diabrotica* × corn, *Nilaparvata* × rice, Agriotes × maize, Agriotes × wheat, *Rhopalosiphum padi* × wheat, *Aphis gossypii* × cotton, *Bemisia tabaci* × cotton.

Our initial dataset was composed of 266 relevant literature references, of which 216 covered technologies that were deemed to be effective—as expressed by the papers’ authors. A particular pest management technology was considered effective if a statistically significant level of control of the target pest was reported. In case one single scientific study comprised multiple effective (and non-effective) tools, these were listed as separate lines in the dataset. From the collated set of references, we extracted those that specifically addressed the evaluation of non-chemical crop protection alternatives, and organized these into six main categories depending upon the primary type of pest management tactic: (1) biological control, (2) cultural (e.g., sanitation, crop rotation, nutrition or water management) or mechanical control, (3) innovative pesticides and application regimes (e.g. attractants, reduced product doses, anti-resistance strategies), (4) host plant resistance, (5) decision-support tools (e.g., monitoring schemes, predictive models, early-warning systems), and (6) other tools such as farming systems adaptations, multi-faceted IPM packages, and others. As the same technology was regularly covered in multiple articles, we consolidated literature records for the expert evaluation: a total of 17 different tools for western corn rootworm, 26 for bird cherry-oat aphid, 25 for wireworms, and 23 for brown planthopper were included in the questionnaire and evaluated by experts. Furthermore, for studies that were exclusively based on field data, we mapped the availability of different technologies within particular geographical areas. Overall, Africa was underrepresented in the dataset.

### Expert evaluation of the alternative tools

Literature references were compiled and tabulated per crop × pest system, and subsequently shared by email with expert scientists and pest management professionals for further commenting and updating. Experts could rank the “readiness” of each management alternative using the following criteria: RESEARCH = at research stage; READY = available for immediate implementation, though not yet adopted by local growers; PRACTICED = adopted and used by (nuclei of) growers in a particular country or region. Furthermore, experts were requested to indicate their perceived importance of two potential “roadblocks” or constraints to broader technology diffusion and uptake, i.e., ENVI = technology is considered ineffective under local environmental conditions; ECONOM = current technology is deemed to be too expensive or not economically viable, thus limiting wider adoption.

Online survey for pest risk assessment

An online survey was made available through a dedicated cloud-based platform (i.e., SurveyMonkey) and shared with the above mentioned global experts. Through this survey tool, experts were able to rank the perceived importance of a given insect herbivore (in a particular crop) and share information on its infestation pressure as related to (locally established) economic thresholds, or other metrics reflective of its economic relevance. Also, through the online survey, data were gathered on the relative extent of neonicotinoid use (i.e., % growers, treated area) and pest management alternatives.

For either evaluation method, feedback was obtained from a total of 16 scientific experts and crop protection professionals, from China, Croatia, Germany, Hungary, Indonesia, Italy, Philippines, Poland, Slovenia, Spain, USA, and Vietnam. Experts provided additional papers, non-peer-reviewed documents and reports, and other information to update our database of pest management alternatives. As no feedback was received for cotton pests, no assessments could be carried out for *A. gossypii* and *B. tabaci*, so cotton was excluded from the further evaluation. No responses were received from scientists in Africa, Latin America and the Caribbean, or in Central Asia.

Given that the collected data are descriptive, results were tabulated to provide a full overview of the evaluated alternatives. Results from the expert evaluation (average number of tools ranked per category) were visualized using polar bars, while the geographical coverage of field-tested alternatives is shown in maps. Lastly, bar charts show the results of the pest risk assessment—as obtained through the online survey.

## Results

### Literature review and expert evaluation of alternative tools

Most literature references were found for *B. tabaci*, while the lowest number of references was recorded for *A. gossypii* and *D. virgifera* (Table 1). Biological control featured prominently as management alternative for all crop × pest systems, ranging from few effective technologies for *N. lugens* to more than half of the alternative management tools for *B. tabaci* (Table 1). Only half of the effective management tools were based on field data, most of which were from Europe for wireworms in maize and winter wheat, from Asia on *N. lugens* in rice, and from Asia and North-America on *B. tabaci* in cotton. In the following sections, we describe findings for the most effective management alternatives.

**Table 1:** Overview of the availability of effective pest management alternatives (number of paper published), as organized for each of the main crop × pest complexes based on the literature review; *SLW* silverleaf white fly, *WCR* western corn rootworm, *BPH* brown planthopper

Categories	Geographic distribution based on the location of the experiments in the papers	Crop and pest						
		Cotton		Maize	Maize, wheat	Rice	Wheat	Total
		Aphid	SLW	WCR	Wireworms	BPH	Aphid	
Biological control (effective 91/total 116)	Asia	3	6			3	1	13
	Australia	1						1
	Europe			2	10		8	20
	North America and Europe						1	1
	North America	2	5		2		1	10
	South America		4					4
	Non field data	6	17	7	1	7	4	42
Cultural or mechanical control (effective 29/total 35)	Asia	1	2			5	2	10
	Europe				4		2	6
	North-America		2	1				3
	Non field data		4	1	3	2		10
DSS (effective 29/32 total)	Africa	1						1
	Asia					2		2
	Australia		1					1
	Europe				10		2	12
	South America	1						1
	Non field data	2		1	3	1	5	12
Innov. pestic. and appl. reg. (effective 28/30 total)	Asia		3		1	1		5
	Europe			1	1			2
	North America			3	1		1	5
	North America, Asia		1					1
	Non field data	2	1	4	2	4	2	15
Other (effective 14/17 total)	Asia	1				3		4
	Europe			1	1		2	4
	North-America		1		2			3
	Non field data		1			1	1	3
Host plant resistance (effective 25/36 total)	Asia		1			1	1	3
	Australia		1					1
	Europe			2			1	3
	North-America			1				1
	Non field data		3	4		6	4	17
Sum		21	54	28	41	36	38	216

**Fig. 1:**
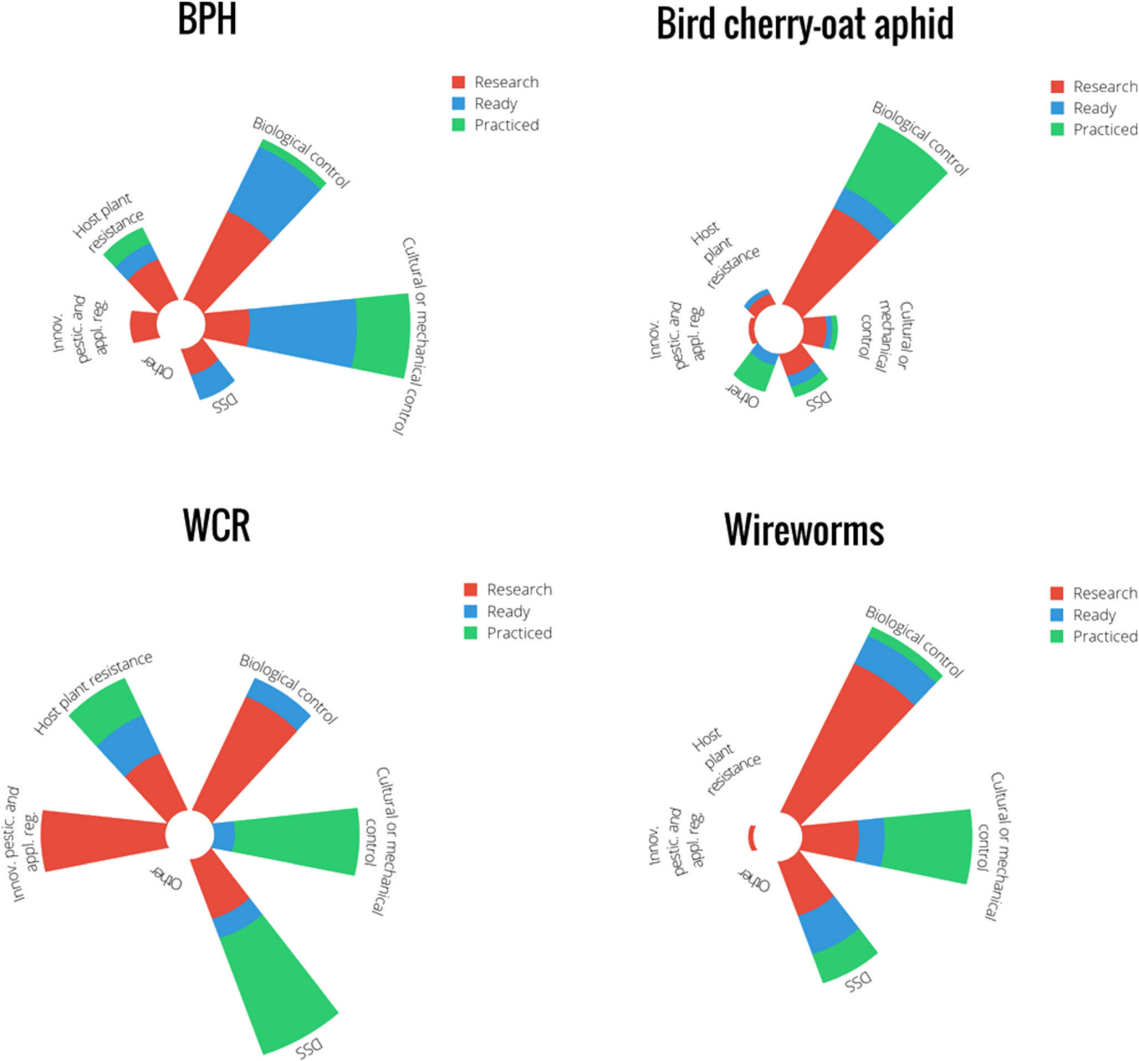
Implementation “readiness” of management alternatives for BPH (brown plant hopper, *Nilaparvata lugens*) in rice, bird cherry-oat aphid (*Rhopalosiphum padi*) in winter wheat, WCR (western corn rootworm *Diabrotica virgifera virgifera*) in maize, and wireworms (*Agriotes* spp*.*) in maize and winter wheat. Relative size of a given section (or arm) within the 4 star-plots reflects the number of literature records covering a particular IPM technology and their respective “readiness” status (i.e., “under research,” “ready,” or “practiced”)

**Fig. 2:**
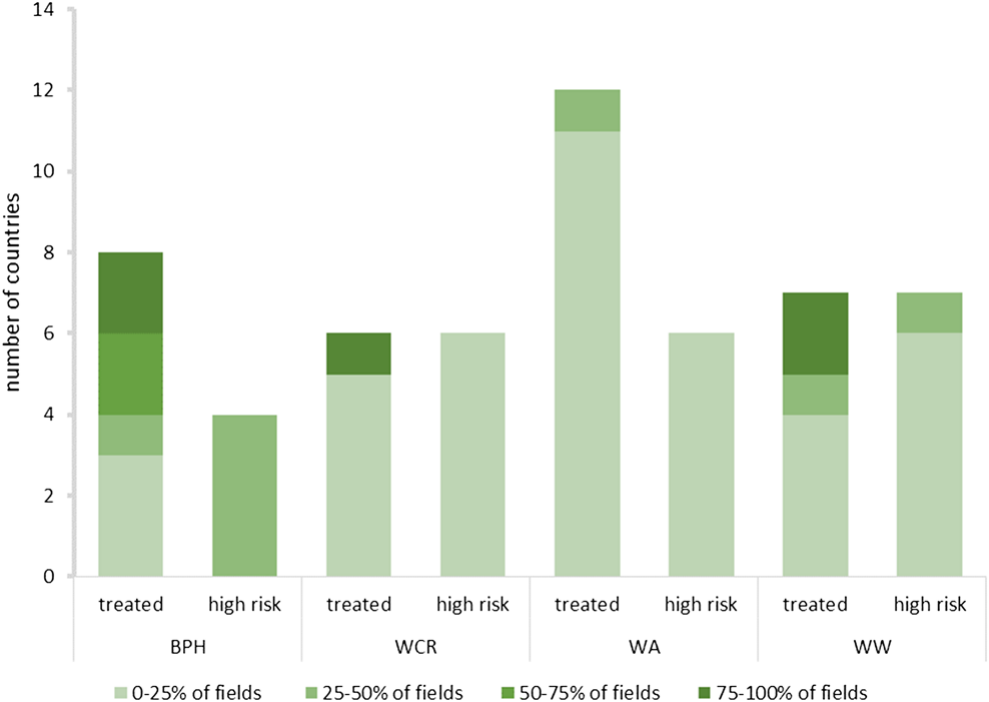
Number of countries with varying share of “high-risk” fields and of neonicotinoid-treated fields for each of the 4 target herbivores, as based on the online survey involving 16 experts. BPH = brown planthopper (foliar and seed treatment), WCR = western corn rootworm (soil insecticide), WA = bird cherry-oat aphid (foliar and seed treatment), WW = wireworm (soil insecticide)

**Fig. 3:**
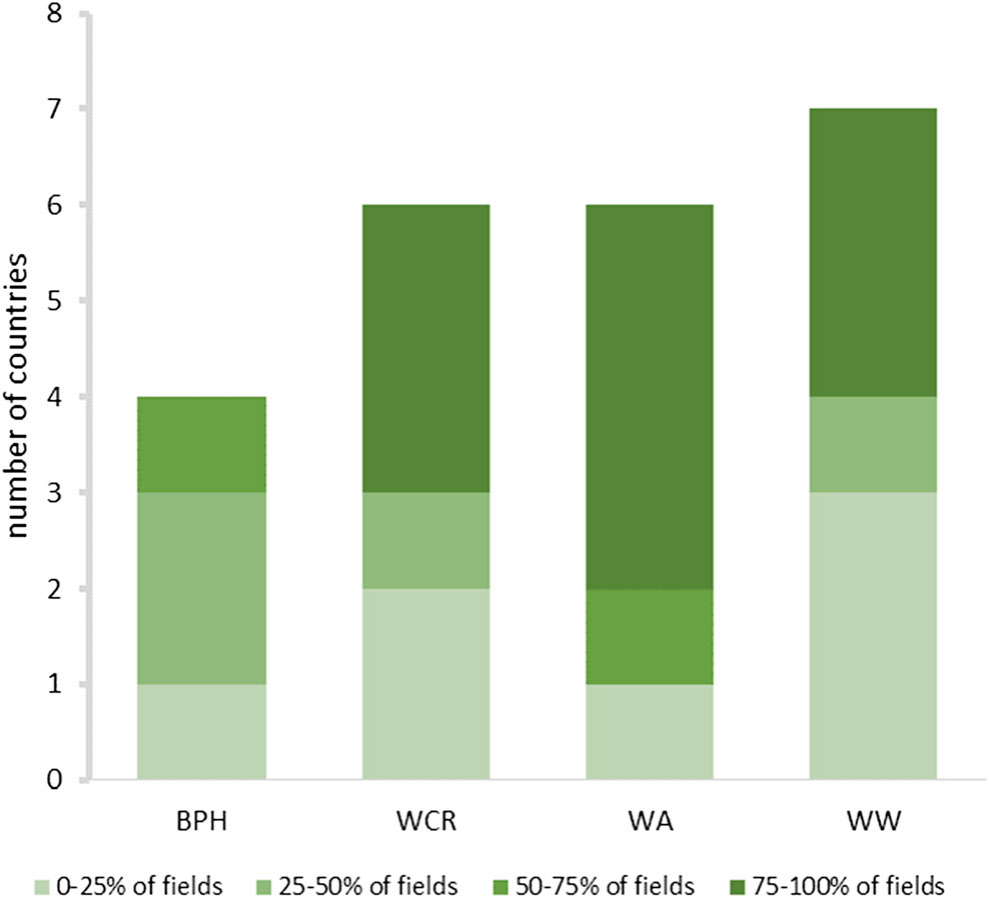
Number of countries indicating varying share (%) of fields in which either of the 4 target herbivores could be effectively managed with (non-chemical) IPM alternatives, as drawn from the online survey with 16 experts. BPH = brown planthopper, WCR = western corn rootworm, WA = bird cherry-oat aphid, WW = wireworm

Approximately half of the tools were only reported to be at a research stage. Biological control was extensively documented in literature records, yet the majority of those were evaluated to be only at a research stage. The highest percent of tools in practice were found for bird cherry-oat aphid control, and primarily included CBC (Conservation Biological Control) through landscape and habitat-level management (Fig. 1). For brown plant hopper control in rice, mostly cultural and mechanical control technologies were put in practice by growers, however only in a geographically limited area (i.e., parts of Vietnam and Indonesia). For wireworm control, cultivation practices and DSS were most practiced. Western corn rootworm control was built around three main pillars: DSS, host plant resistance and cultural practices (i.e., crop rotation). The online survey further revealed low perceived risk of western corn rootworm in maize, aphids in winter wheat and wireworms in maize and in winter wheat (Fig. 2), thus creating room for field-level evaluation and grower adoption of non-chemical alternatives (Fig. 3). For western corn rootworm and wireworms, several countries reported 75–100% adoption levels of neonicotinoids in their respective maize and wheat crops (Fig. 3). Though brown planthopper poses low to intermediate risks in rice (i.e., 25–50%), rice fields are routinely treated with neonicotinoids (Figs. 2 and 3). Below we discuss the main alternatives to neonicotinoids in further detail.

### Biological control

For bird cherry-oat aphid control in winter wheat, nine different CBC tools were reportedly practiced in Italy, Hungary, Spain and USA (Tables 2 and 3). In winter wheat, aphid biological control was primarily at the research stage, and few countries mentioned economic or environmental roadblocks for subsequent grower adoption. For wireworm control in maize and winter wheat, biological control is primarily at the research stage and only two technologies—using entomopathogenic fungi—are being practiced in Germany and the USA (Tables 5 and 6). Most countries reported the existence of environmental and economic roadblocks for the field-level use of biopesticides and nematodes, while landscape and habitat management tools were considered “ready for implementation” in Slovenia and Hungary. Few countries indicated studies at research stage, and only nematodes might be ready for use in Germany. For western corn rootworm management in maize, no biological control technologies are being implemented yet—possibly due to locally perceived economic and environmental barriers, or technology related issues (Table 7).

**Table 2:** Technology “readiness” of pest management alternatives for bird-cherry oat aphid (*Rhopalosiphum padi*) in winter wheat, and locally perceived obstacles for their further farmer-uptake and diffusion (based on the expert consultation) - first part

Categories	Sub-categories	IPM alternatives identified	Evaluation criteriaa			Roadblocks^b^	
			RESEAR	READY	PRACTIC	ENVI	ECON
Biological control	Biopesticides	Phenolic acid extracts from leaves of black currant (*Ribes nigrum* L.), sour cherry (*Prunus cerasus* L.) and walnut (*Juglans regia* L.), and from the green husks of walnut have insecticidal effect (Chrzanowski et al. 2012)					HU
	Biopesticides	Garlic oil blend can be used for aphid control (Zhou et al. 2013)	IT				HU
	Systemic defense priming	DIMBOA increases aphid susceptibility to electrophilic agents and insecticides (Mukanganyama et al. 2003)	HU				
	CBC, predators	Diverse arthropod predator community, including *Pardosa* and *Enoplognatha* spiders, reduces aphids to low densities (Kuusk et al. 2008, Opatovsky et al. 2012)	HU,US		ES,IT		
	CBC, parasitoids	Leguminose flower strips allow (non-pest) aphid species to build up, thus increasing parasitoid populations and enhancing in-field biological control (Langer and Hance 2004)	HU,ES, SI,US		IT	ES	
	CBC, predators and parasitoids	Both foliage- and ground-foraging natural enemies reduced aphid numbers by 90-93%, whereas ground-foraging predators alone achieved a 18-40% reduction (depending upon width of field margin) (Holland et al. 2008)	HU,US		IT		
	CBC, fungi	Insect-killing fungi infect aphids on its winter host bird cherry (Nielsen and Steenberg 2004)	HU		IT	ES	
	CBC	Soil-inhabiting decomposer communities (incl. Collembola) benefit wheat growth and slow aphid reproduction (Schütz, Bonkowski, and Scheu 2008)	HU		ES		
	Landscape effect	Lower aphid numbers under organic farming schemes (at the time of wheat flowering). Complex landscapes provided more overwintering sites, alternative hosts, and nectar sources and boosted parasitism levels (Roschewitz et al. 2005)	ES, US	HU	IT		US
	Landscape effect	Complex landscapes had 46% higher rates of pest control than simplified ones (i.e., dominated by cultivated land) (Rusch et al. 2016)	US	HU	IT	SI	

^a^PRACT = is widely used in the country; RESEAR = at research state only; READY=IPM alternative available for immediate implementation; ^b^frequently listed roadblocks, preventing technology diffusion: ENVI = it would not be effective under the environmental conditions of the country; ECON = deemed too expensive—so it is not widely adopted; ES (Spain), IT (Italy), SI (Slovenia),HU (Hungary), US (United States), DE (Germany), HR (Croatia)

**Table 3:** Technology “readiness” of pest management alternatives for bird-cherry oat aphid (*Rhopalosiphum padi*) in winter wheat, and locally perceived obstacles for their further farmer-uptake and diffusion (based on the expert consultation) - second part

Categories	Sub-categories	IPM alternatives identified	Evaluation criteriaa			Roadblocks^b^	
			RESEARCH	READY	PRACTICED	ENVI	ECON
Biological control	Field margin and landscape effect	Landscapes with abundant field margins and perennial crops had low rates of aphid establishment. After establishment, there was no difference in ground-living enemy impact on R. padi population growth rate between farming systems, but impact was greater in landscapes where arable land was contiguous. Natural pest control declines with distance from the crop edge. (Östman, Ekbom, and Bengtsson 2001)	ES, US	HU	IT	SI	
	Landscape effect	Nearby presence of grassland and hedges decreased aphid numbers; with woody habitats enhancing hoverflies and hedges benefiting parasitism (Alignier et al. 2014)	ES,SI	HU	IT		
Cultural or mechanical control	Inter-cropping	8-2 or 8-4 row alternation with oilseed rape (OSR) lowered aphid densities; wheat-OSR intercrops also conserved more natural enemies than monocultures and partial resistance of wheat cultivar had synergistic effects on parasitoids of aphids (Wang et al. 2009)	IT,US				
	Inter-cropping	Wheat-garlic intercropping had lower aphid densities, and garlic volatiles attracted natural enemies such as ladybirds and parasitoids (Zhou et al. 2013)					
	Nutrition management	Reduction in N-fertilizer application can lower aphid adult weight, fecundity, longevity development time. Optimize N fertilization (Aqueel and Leather 2011)	IT,US	ES	HU		
DSS	Timing	Early-sown plots had higher yields; insecticide applications in spring-sown plots should be discouraged and properly timed depending upon patterns in crop phenological stage, aphid colonization and infectivity (Mann et al. 1997)	IT	ES	HU,US		
	Model	GETLAUS is a model for simulating aphid population dynamics as related to crop development and in-field natural enemy populations; it allows visualizing the effect of pesticides on aphids, beneficial insects and yield losses (Gosselke et al. 2001)	HU				

^a^PRACTICED = is widely used in the country; RESEARCH = at research state only; READY=IPM alternative available for immediate implementation; ^b^frequently listed roadblocks, preventing technology diffusion: ENVI = It would not be effective under the environmental conditions of the country; ECON = deemed too expensive - so it is not widely adopted; ES (Spain), IT (Italy), SI (Slovenia),HU (Hungary), US (United States), DE (Germany), HR (Croatia)

**Table 4:** Technology “readiness” of pest management alternatives for bird-cherry oat aphid (*Rhopalosiphum padi*) in winter wheat, and locally perceived obstacles for their further farmer-uptake and diffusion (based on the expert consultation) - third part

Categories	Sub-categories	IPM alternatives identified	Evaluation criteriaa			Roadblocks^b^	
			RESEARCH	READY	PRACTICED	ENVI	ECON
DSS	Model	APHIDSim and other models predict in-field aphid population dynamics based upon natural enemy abundance, climatic conditions, pesticide use, crop, wind speed, wind direction, spatial factors, population dynamics (Wu et al. 2014, Piyaratne, Zhao, and Meng 2013, Rossing, Daamen, and Hendrix 1994, Fabre et al. 2010, Parry, Evans, and Morgan 2006)	HU				
	Model	Cereal Aphids Expert System (CAES) enables aphid identification and provides key agro-ecological and management information, including alternative host plants, damage, injury level, control tactics. Can be consulted through phone and videotext (Gonzalez-Andujar, Garcia-de Ceca, and Fereres 1993)	HU	ES	IT		
Other	Organic farming system	Organic farms had higher impact of natural enemies and lower aphid densities (during wheat flowering) (Östman, Ekbom, and Bengtsson 2001, Roschewitz et al. 2005)		HU	IT,SI		
	IPM	Pest management package composed of chemical control with decision rules, plant resistance, biological control, and farming practices (Dedryver, Le Ralec, and Fabre 2010)		HU	ES,IT,SI, US		
Innov. pestic. and appl. reg.	Seed dressing	Soaking seeds in thiamine (Vitamin B1) or addition of thiamine to nutrient solutions reduces aphid growth and reproduction (Hamada and Jonsson 2013)	HU				
Host plant resistance	GMO	Transgenic expression of Hpa1_10–42_ in wheat induces resistance (Xu et al. 2014)				HU	HU
	Varietal resistance	Plant genotype modulated adult body weight, fecundity and development rate (Zhang et al. 2016, Aqueel and Leather 2011)	US	HU			
	Varietal resistance	Variety Xiaoyan 22 has a thicker cell wall and inner tissue during seedling stage to hinder aphids in feeding on the plants (Hu et al. 2008)				HU	
	Varietal resistance	Two dominant, aphid resistance genes encode NBS-LRR proteins involved in the specific recognition of aphids to date, however resistant breaking biotypes in plant-aphid system has occurred (Dogimont et al. 2010)	HU				

^a^PRACTICED = is widely used in the country; RESEARCH = at research state only; READY=IPM alternative available for immediate implementation; ^b^frequently listed roadblocks, preventing technology diffusion: ENVI = it would not be effective under the environmental conditions of the country; ECON = deemed too expensive - so it is not widely adopted; ES (Spain), IT (Italy), SI (Slovenia),HU (Hungary), US (United States), DE (Germany), HR (Croatia)

**Table 5:** Technology “readiness” of pest management alternatives for wireworms (*Agriotes* spp.) in winter wheat and in maize, and locally perceived obstacles for their further farmer-uptake and diffusion (based on the expert consultation) - first part

Categories	Sub-categories	IPM alternatives identified	Evaluation criteria			Roadblocks^b^	
			RESEARCH	READY	PRACTICED	ENVI	ECON
Biological control	Biopesticides	Extracts from defatted seed meals of *Brassica carinata* sel. ISCI 7 had insecticide effect (Furlan et al. 2010)	DE, IT,SI,HU			DE	HU
	Biopesticides	Aqueous solution of chopped fresh *Brassica juncea* leaves decreased population (Furlan et al. 2010)	DE, IT,SI,HU			DE	
	Bacteria	*Rickettsiella agriotidis* can be used in insecticidal sprays (Kleespies et al. 2013, Leclerque et al. 2013)	IT,HU				
	Nematodes	*Heterorhabditis bacteriophora*, *Steinernema carpocapsae* (at a dose of 50 or 100 IJs/cm2) controlled *A. obscurus* larvae (Kleespies et al. 2013, Ansari, Evans, and Butt 2009, Rahatkhah et al. 2015, Morton and Garcia-del-Pino 2017)	IT,ES,US			DE, HU	HU
	Fungi	*B. bassiana* (Balsamo) has an insect-killing effect (Leclerque et al. 2013, Kleespies et al. 2013, Ansari, Evans, and Butt 2009, Kabaluk 2014)	ES,US	DE, US,IT,SI		HU	HU
	Fungi	*M. anisopliae* strains V1002 and LRC181A has insecticide effect (Ansari, Evans, and Butt 2009)	IT,SI,ES,US	DE, US			
	Fungi	*Metarhizium brunneum* Petch isolate LRC112 conidia as seed dressing or as dust (Kabaluk 2014)	IT,SI,ES,US				
	Fungi	*M. brunneum* F52, *Beauveria bassiana* GHA, *M. robertsii* DWR 346 applied as in-furrow granular or soil band-over-row drench (Reddy et al. 2014)	IT,SI,ES,US				
	Field margins	Avoid the establishment of grassy field margins (Hermann et al. 2013)	DE,US	SI		HU	
	Landscape management	Hedges or cultivated crops at the field border decreases wireworm attack, while grassland increases wireworm problems (Saussure et al. 2015)	DE,IT,SI	HU			
Cultural or mechanical control	Alternative crops	Crop diversification can benefit wireworm control; mustard, cabbage, French marigold, clover and flax are less susceptible to attack, while pea and bean plants tolerate attack (Griffiths 1974)	DE	ES, IT	HU		
	Crop rotation	Rotation with meadows increases wireworm damage (Furlan, Contiero et al. 2017, Poggi et al. 2018, Saussure et al. 2015)			DE,HR,IT,HU,ES,US,SI		
	Crop rotation	Rotation with less susceptible crops and biocidal crops, such as oilseed rape (Furlan et al. 2009, Ritter and Richter 2013)	US		DE,IT,HU,ES, SI	HR	

^a^PRACTICED = is widely used in the country; RESEARCHED = at research state only; READY=IPM alternative available for immediate implementation; ^b^frequently listed roadblocks, preventing technology diffusion: ENVI = it would not be effective under the environmental conditions of the country; ECON = deemed too expensive—so it is not widely adopted; ES (Spain), IT (Italy), SI (Slovenia),HU (Hungary), US (United States), DE (Germany), HR (Croatia)

**Table 6:** Technology “readiness” of pest management alternatives for wireworms (*Agriotes* spp.) in winter wheat and in maize, and locally perceived obstacles for their further farmer-uptake and diffusion (based on the expert consultation) - second part

Categories	Sub-categories	IPM alternatives identified	Evaluation criteriaa			Roadblocks^b^	
			RESEARCH	READY	PRACTICED	ENVI	ECON
Cultural or mechanical control	Intercropping	Grass and legume are preferred by wireworm larvae (as compared to maize and forbs); their inclusion as intercrops can lower wireworm damage (Schallhart et al. 2012)	DE,IT, US	HU			
	Tillage timing	Meadow plowing just before seeding (Furlan et al. 2020)	IT				
	Trap crop	Cultivating trap crop, such as pea and lentil (Ritter and Richter 2013)	HR,IT	HU			
	Nutrition management	Application of CaCN2 can have a non-toxic yet repellent effect on local wireworm populations (Ritter et al. 2014)	HU	IT	DE	DE	
	Tillage	Wireworm population negatively correlates with the number of tillage applications, depending on weather conditions (Saussure et al. 2015)	DE,IT, US	SI	HU,ES		
DSS	Monitoring	Establishment of bait traps, baited with wheat seedlings (Furlan 2014, Furlan, Contiero et al. 2017, Parker 1994, 1996, van Herk and Vernon 2013)	HR,IT	SI,HU	US		
	Monitoring	Trap made of durable plastic, baited with pheromone lures (Jung et al. 2014)	HU	HR,SI	DE,US,IT		ES
	Monitoring	Collection of larvae with bait traps–instead of adults with pheromone traps– can help assess risk of wireworm attack (Benefer et al. 2012)	HR,HU,US	SI	IT	DE	IT
	Abiotic factors	Altitude, precipitation, mean temperature, and soil pH, water balance, humus content and texture are all risk factors for wireworm attack (Furlan et al. 2017, Hermann et al. 2013)	DE,HR	HU	IT,HU		
	Threshold level	>1, 5 and 2 larvae/trap for *A. brevis*, *A. ustulatus* and *A. sordidus* respectively. Thresholds apply for: (1) bare soil with no alternative food sources; (2) average soil temperature 10 cm beneath the surface of >8 °C for 10 days; (3) soil moisture near to field water capacity (Furlan 2014)	IT	HU			
	Model	SIMAGRIO-W is a simulation model that determines risk of wireworm attack (Jung et al. 2014)	DE,IT	HU			
Innov. pestic. and appl. reg.	Attract and kill	Attraction to an artificial CO_2_-emitting source, using baker’s yeast in combination with *M. brunneum* conidia for wireworm infection (Brandl et al. 2017, Gfeller et al. 2013, Barsics et al. 2017)				HR, ES,US	
	Physical barrier	A portable trench barrier, composed of an extruded PVC plastic trough can prevent *A. obscurus* field colonization (Vernon and van Herk 2013)	HU				IT,HU,ES

^a^PRACTICED = is widely used in the country; RESEARCH = at research state only; READY=IPM alternative available for immediate implementation; ^b^frequently listed roadblocks, preventing technology diffusion: ENVI = it would not be effective under the environmental conditions of the country; ECON = deemed too expensive - so it is not widely adopted; ES (Spain), IT (Italy), SI (Slovenia),HU (Hungary), US (United States), DE (Germany), HR (Croatia)

**Table 7:** Technology “readiness” of pest management alternatives for western corn rootworm (*Diabrotica virgifera virgifera*) in maize, and locally perceived obstacles for their further farmer-uptake and diffusion (based on the expert consultation)

Categories	Sub-categories	IPM alternatives identified	Evaluation criteriaa			Roadblocks^b^	
			RESEARCH	READY	PRACTICED	ENVI	ECON
Biological control	Biopesticides	Turmeric extracts (*Curcuma longa*) are repellent (Brandl et al. 2016)	DE				
	Nematodes	*Heterorhabditis bacteriophora*, *H. megidis*, *Steinernema feltiae* have all proven to be effective for WCR control (Pilz et al. 2009)	SI	DE		HU	DE,US
	Nematodes	Root cap exudates of green pea and maize can enhance efficacy of *Heterorhabditis megidis* in the field (Jaffuel, Hiltpold, and Turlings 2015)	DE			HU	US
	Ind. self. def.	Induced self-defense with jasmonic acid (Capra et al. 2015, Stenberg et al. 2015, Erb, Glauser, and Robert 2012)	HU				
	Class. bio. cont.	*C. compressa* introduction into invaded areas (Toepfer et al. 2009)	HU,DE				
Cultural or mechanical control	Crop rotation	Crop rotation, 100% of rotation is not needed (Szalai et al. 2014)		DE	HU,HR,SI,DE,PL,US, IT		
	Winter cover crop	an autumn-planted and spring-killed grass cover crop enhanced abundance of predator populations and led to significantly less root damage in the subsequent maize crop (Lundgren and Fergen 2011)					
DSS	Monitoring	Pheromone traps – sticky sheet and non-sticky container traps (added by the countries		HR	HU,SI,DE,PL,US		
	Model	% of fields with WCR above thresh. level in a region depends (i) on rotation rate (ii) on the pop. growth rate of WCR at low pop. density (Szalai et al. 2014)	HR,DE,US		HU, IT		
	Model	Decision-support model, based upon degree-day, to estimate hatching time and adult emergence (add. By SI)			SI		
Host plant resistance	GMO	*Bt* maize		HU	US		
	GMO	RNA interference (RNAi), amylase inhibitors from common bean and soybean cysteine proteinase inhibitor N (soyacystatin N, scN). (Fishilevich et al. 2016)	HU,PL,US	HU			
	Refuge establishment	In GMO-based systems, refuges along with WCR-resistant corn (add. US)			US		
Innov. pestic. and appl. reg.	Attract and kill	Powdered buffalo gourd, *C. foetidissima*, corn seedling volatiles, CO_2_, extracts of germ. corn (Schumann, A.Patel, and Vidal 2013, Cossé and Baker 1999, Schumann et al. 2014, Hibbard and Bjostad 1988, Hibbard and Bjostad 1990)	HU,DE				
	Attract and kill	α-terpineol, β-caryophyllene, hydroxamic acids, long-chain free fatty acids are attractive (Hammack 1996, 2001, Hibbard, Bernklau, and Bjostad 1994, Xie et al. 1992, Hibbard and Bjostad 1990)	HU,DE				
	Mating disrupt.	Pheromone-based mating confusion (add. by SI)	SI				
	Attract and Kill	CO_2_-releasing capsules and a *Metarhizium* strain can be combined in an Attract & Kill scheme (add. by DE)			DE		

^a^PRACTICED = is widely used in the country; RESEARCH = at 8research state only; READY=IPM alternative available for immediate implementation; ^b^frequently listed roadblocks, preventing technology diffusion: ENVI = it would not be effective under the environmental conditions of the country; ECON = deemed too expensive—so it is not widely adopted; ES (Spain), IT (Italy), SI (Slovenia),HU (Hungary), US (United States), DE (Germany), HR (Croatia), PL (Poland)

For brown planthopper control, several biological control tools have been described in the literature. Yet, only one landscape management tool was practiced in Indonesia, most options are ready for implementation in Vietnam, and remain at the research stage in Papua New Guinea (Table 8). CBC—particularly the use of flower strips to enhance in-field populations of parasitoids—has been implemented in southern Vietnam, and has been extensively researched at experimental stations in Thailand, China and the Philippines and in farmers’ fields in China. In Vietnam, its farm-level adoption may potentially accelerate.

**Table 8:** Technology “readiness” of pest management alternatives for brown planthopper (*Nilaparvata lugens*) in rice, and locally perceived obstacles for their further farmer-uptake and diffusion (based on the expert consultation) - first part

Categories	Sub-categories	IPM alternatives identified	Evaluation criteriaa			Roadblocks^b^	
			RESEAR	READY	PRACT	ENVI	ECON
Biological control	Biopesticides	Jasmonic acid induces systemic defenses, impacting BPH (Senthil-Nathan, Kalaivani, et al. 2009)	PG				
	Biopesticides	Active compounds in the extracts of persimmon (*Diospyros kaki*) roots have insecticidal effects (Jeon et al. 2011)	PG, ID				
	Biopesticides	ar-turmerone, as obtained from the rhizome of turmeric (*Curcuma longa*), has insecticidal properties (Lee et al. 2001)	PG				
	Biopesticides	Methyl-eugenol has repellent and both systemic and contact insecticidal activities against BPH, while low toxicity for natural enemies (Xu et al. 2015)	PG, VN4	VN1			
	Biopesticides	Extracts of dried roots of the Chinese medicinal herb *Euphorbia kansui* showed pesticidal activity against BPH (Dang et al. 2010)	PG	VN1			
	Biopesticides	Neem seed powder and leaves of crown flower (*Calotropis gigantean*), used as soil amendments, improved plant resistance (Senthil-Nathan, Choi, et al. 2009)	PG, VN4	VN1			
	Predator	The climbing perch (*Anabas testudineus*) can enhance BPH biological control, within rice-fish production systems (Cao Quoc et al. 2012)		VN3		VN4	
	CBC	Frogs consumed significant numbers of rice pests, with levels of pest consumption highest during the dry season (Khatiwada et al. 2016)	ID	VN1, VN2		VN4	VN4
	CBC	*Anagrus nilaparvatae* is a naturally occurring key parasitoid of BPH, though its efficacy can be determined by climatic conditions (Ma, Peng, and He 2012)	PG			VN3	VN4
	Landscape management	Sustaining diverse landscape mosaics, especially in smallholder rice production systems, can reduce pesticide applications by 75% (Westphal et al. 2015, Gurr et al. 2016)		VN1,PG	ID	VN3	
Cultural or mechanical control	Intercropping	Intercropping with corn versus monoculture lowered BPH populations (Yao et al. 2012, Lin et al. 2011)	PG			VN3	
	Nutr. management	Optimization of nitrogen fertilization, through nutrient management, can reduce BPH populations in rice crops (Rashid, Jahan, and Islam 2016)	VN4	VN2, PG, VN3	VN1, VN2, ID		
	Nutr. management	Potassium fertilization contributed to an enhanced tolerance of plants to BPH (Rashid, Jahan, and Islam 2016)	VN4	VN1,VN2,VN3		PG	
	Nutr. management	Addition of silicon enhances rice plant resistance to BPH (Rashid, Jahan, and Islam 2016)		VN1, PG,VN3	VN2		
	Water management	Drought and accompanied water stress induces pest outbreaks, with BPH nymphs and adults preferring stressed plants (He et al. 2015)	PG, VN4	VN1, VN2, VN3	VN2, VN4		

^a^PRACTICED = is widely used in the country; RESEARCH = at research state only; READY=IPM alternative available for immediate implementation; ^b^frequently listed roadblocks, preventing technology diffusion: ENVI = it would not be effective under the environmental conditions of the country; ECON = deemed too expensive—so it is not widely adopted; ID (Indonesia), PG (Papua New Guinea), VN1, VN2,VN3, VN4 (Vietnam)

Under field conditions, micro-organisms and biopesticides are perceived to have more potential for use than macro-organisms because they are easier to store and transport, and can be bulked up under laboratory conditions. Nematodes have been used to control soil pests such as WCR in maize under laboratory and field conditions (Kurtz et al. 2008; Toepfer et al. 2010) and wireworms under laboratory conditions (Ansari et al. 2009). In arable crops, the following species of entomopathogenic fungi have been tested: *Beauveria bassiana, Metarhizium* spp. and *Lecanicillium lecanii* (*Verticillium*) (Kabaluk 2014; Kim and Kim 2008; Ritter and Richter 2013; Ansari et al. 2009). Fungal applications may be combined with augmentative releases of predators (i.e. *Orius laevigatus* (Down et al. 2009)) or parasitoids (Lazreg et al. 2009), and several of these organisms can contribute significantly to *B. tabaci* control (Antony et al. 2004; Bellamy et al. 2004; Hoelmer 2007; Viscarret and López 2004; Yang and Wan 2011).

Certain plant-produced compounds can prolong the shelf-life of beneficial entomopathogenic nematodes in *Diabrotica* management (Jaffuel et al. 2015). Other compounds have repellent or insecticidal effects, and their extracts can be used as biopesticides. Several biopesticides are also fully compatible with natural biological control, e.g., extracts of *Ruta chalepensis*, *Peganum harmala* and *Alkanna strigosa* inflict levels of *B. tabaci* mortality similar to imidacloprid without negatively affecting its parasitoid *Eretmocerus mundus* (Al-Mazra’awi et al. 2009).

Naturally occurring arthropod predators and parasitoids play a central role in regulating pest populations in arable crops, and their active in-field conservation can constitute a desirable, cost-effective means of pest control (e.g., (Landis et al. 2000, Naranjo et al. 2015, Shields et al. 2019, Veres et al. 2013). Predaceous spiders consume large numbers of aphids and *B. tabaci* (Choate and Lundgren 2015; Kuusk et al. 2008) and conservation measures can lower pest population numbers, associated feeding damage and pest-inflicted yield losses—especially in systems where there is little concern about insect-mediated virus transmission (Naranjo 2001). CBC schemes involve a deliberate suspension (or drastic reduction) of pesticide applications, and the deployment or preservation of in-field shelters, nectar, alternative prey/host items, and pollen to support resident natural enemy communities (so-called SNAP; (Gurr et al. 2017)). Many of these interventions can be laborious and involve added costs, but the returns on investment can be high and should appeal to growers (Naranjo et al. 2015). Ample CBC research has been conducted for several of the target pests, and this increased research attention is warranted. The spatio-temporal availability of certain crop and non-crop habitats can impact field populations of pests and improve their associated natural enemies (Veres et al. 2012; Burgio et al. 2006), although landscape-level impacts can be inconsistent and sometimes difficult to predict (Karp et al. 2018). Hence, interventions have to be carefully chosen and science based to enhance (or sustain) natural enemy populations while counteracting pest attack. For example, certain host plants that are beneficial for natural enemies—e.g., ragweed for *B. tabaci*—can also serve as alternative hosts that favor pest immigration (Naveed et al. 2007; Zhang et al. 2014).

The manipulation of rhizosphere interactions and associated plant defenses can be a lucrative option to further enhance CBC, specifically against soil-dwelling pests (such as wireworms). The stimulation of plant defenses, e.g., by integrating plant mutualists into the standing crop (i.e., beneficial microbes, fungi or entomopathogenic nematodes), by directly manipulating soil organic matter, edaphic fauna and soil fertility or by—indirectly—altering crop rotation sequences can provide important feedback mechanisms that boost pest control or enhance natural enemy abundance (Johnson et al. 2016; Wyckhuys et al. 2017). Resistance priming—through silicon amendments, or EPNs—also offers opportunities for management of sap-feeding pests such as BPH and *B. tabaci* (An et al. 2016; Yang et al. 2017). CBC can equally involve the promotion of entomophthoralean fungi, i.e. by enhancing fungal infection of *R. padi* through feeding on its winter host bird cherry (Nielsen and Steenberg 2004) as well as endophytic entomopathogens on *A. gossypii* (Gurulingappa et al. 2010). Besides invertebrates, vertebrates can assume an important role in the biological control of several of the target pests. Frogs, fish and ducks can consume large numbers of rice pests, including planthoppers (Khatiwada et al. 2016; Zou et al. 2017; Sheng-miao et al. 2004); for WCR and wireworms, birds act as key predators and can suppress field populations (Bollinger and Caslick 1985; Sheng-miao et al. 2004) and rodents possibly engage in larval predation (Tschumi et al. 2018). A phase-out of neonicotinoid use is key to safeguard and fully exploit these vertebrate-mediated pest control services (e.g., (Humann-Guilleminot et al. 2019b). For example, Gurr et al. (2016) have indicated that pest suppression in ecologically engineered rice fields in China was greatest where farmers suspended the use of chemical insecticides. Different trophic levels can also play a role, with aphid suppression related to Collembola-mediated changes in nitrogen resource allocation and wheat crop growth (Schütz et al. 2008).

CBC carries ample potential in the management of target pests in our four focal arable crops. However, the success of CBC will be related to the effective communication to growers of ecological concepts and encouragement of adoption through participatory research or because of recognized economic advantages from crop diversification (e.g., producing sesame on rice bunds). Such features have encouraged the rapid adoption of ecological engineering for planthopper management in Asia (Westphal et al. 2015; Gurr et al. 2016; Horgan et al. 2016). However, for many crop × pest systems, comprehensive evaluations of natural enemy impacts on pest populations have not been carried out and key information is thus absent to guide the development of habitat manipulation schemes.

The scientifically guided introduction of specialist natural enemies for control of invasive pests is a powerful and self-propelling method of biological control (Wyckhuys et al. 2019b) that carries potential for the management of several target pests. For *A. gossypii*, introductions have been made of several natural enemies—including aphidiine and aphelinid wasps, and syrphid flies—in the Pacific islands (Waterhouse 1998). Opportunities may also exist to employ non-native natural enemies for the control of WCR in its invaded range in Europe (Kuhlmann and van der Burgt 1998; Toepfer et al. 2009).

### Cultural or mechanical control

Crop husbandry or cultural practices such as crop rotation, or adapted fertilisation and water management, have received ample attention for the control of silverleaf whitefly in cotton, wireworms in maize and winter wheat, and brown planthopper in rice (Table 1). Experts signaled that various cultural practices are used against brown planthopper, western corn rootworm, and wireworm in the above crops (Fig. 1). For wireworm, crop rotation measures are either “in practice” or “ready for implementation” in all countries, except for USA (Table 5). Soil fertility management was in practice—with certain environmental constraints—in Germany, and ready to use in Italy. Tillage is commonly practiced in Hungary and in Spain, and ready for implementation in Slovenia, while its effects are still being researched in Germany, Italy and the USA (Esser et al. 2015). For brown planthopper in rice, plant nutrition and water management are either put in practice or ready for implementation, in Vietnam and Papua New Guinea (Table 8). This includes the reduction of nitrogen application rates as part of Vietnam’s “three reductions—three gains” (3R3G) campaign (Horgan 2018). In Indonesia, local government authorities can delay farmer access to irrigation water to enforce fallow periods and abate severe brown planthopper outbreaks (Horgan & Stuart, personal observation). For aphids in wheat and western corn rootworm in maize, few agronomic tools were recorded (Tables 2 and 7), however crop rotation is widely practiced for western corn rootworm control (Fig. 1). Nutrition management is in practice in Hungary and ready for implementation in Spain for aphid control (Table 2). Meadow plowing timing, just before maize seeding, is an effective tactic to prevent wireworm damage to maize (Furlan et al. 2020). Crop rotation can assist with pest control in multiple ways, e.g., by providing a habitat in which pest species are unable to successfully complete their lifecycle (e.g., by replacing host plants with non-hosts, or by creating conditions that disproportionately favor a pest’s natural enemies). Diversification measures can equally be implemented within a given crop, by concurrently establishing a companion crop through intercropping, strip-cropping or relay cropping. These kinds of system-level adaptations have been tested for all target pests.

Though regularly overlooked by pest management professionals, plant nutrition, and water management can be important levers within system-level IPM strategies, and this has received some attention for all pests except WCR. For aphids, BPH and SLW, ecological fitness significantly increased with enhanced levels of nitrogen fertilization of the host crop (Aqueel and Leather 2011; Lu et al. 2004; Crafts-Brandner 2002; Bi et al. 2003). On the contrary, for BPH, additions of potassium and silicon can increase resistance of rice plants and thus lower pest-inflicted production losses (Rashid et al. 2016; Liu et al. 2013; He et al. 2015). Balanced fertilization schemes and the incorporation of organic matter in paddy rice systems can further enhance the build-up of natural enemy populations and boost pest control (Settle et al. 1996).

### Innovative pesticides and application regimes

Novel insecticides or innovative application methods are available for most target pests, and > 50% records report their in-field evaluation (Table 1), primarily in Asia and North America. For bird cherry-oat aphid, innovative insecticides were not deemed relevant (Table 4). For wireworm, attract and kill, mass trapping, or physical barriers were reported, yet environmental or economic roadblocks to their implementation were regularly mentioned (Table 6). Innovative chemical-based approaches for western corn rootworm control in maize (attract and kill, mating disruption, protein biopesticide) and for brown planthopper in rice (reduced dose, innovative insecticides, anti-resistance strategies) were only at the research stage and are considered in few countries (Tables 7 and 9).

**Table 9:** Technology “readiness” of pest management alternatives for brown planthopper (*Nilaparvata lugens*) in rice, and locally perceived obstacles for their further farmer-uptake and diffusion (based on the expert consultation) - second part

Categories	Sub-categories	IPM alternatives identified	Evaluation criteriaa			Roadblocks^b^	
			RESEARCH	READY	PRACICEDT	ENVI	ECON
DSS	Threshold	Thresholds for BPH should be corrected for pest levels of the rice leaffolder (*Cnaphalocrocis medinalis*) (Zheng et al. 2007)		VN2		PG, VN3	
	Model	BPH occurrence system is a chaotic system and predictable time-scale of 79-175 days (Ma, Ding, and Cheng 2001)	PG, VN4	VN3			
	Model	ENSO (El Nino/Southern Oscillation) model indices can be indicative of early-season BPH immigration (Xian et al. 2007)	PG	VN3			
Innov. pestic. and appl. reg.	Innovative insecticide	Validamycin plays a regulatory role in BPH chitin synthesis, thus interfering with the molting process from larvae to adult (Tang et al. 2017)—note field level use of antibiotics raise environmental issues	VN1, PG				
	Innovative insecticide	Feeding of dsRNAs of overexpressed nuclear receptor (NR) genes caused significant nymph mortality (Xu et al. 2017)	PG				
Host plant resistance	Res. var.	Several wild rice species have proven resistance to BPH	PG, VN3				
	Res. Var.	Six rice varieties from South Asia were consistently resistant to BPH, but many varieties with known resistance genes had only weak resistance due to planthopper adaptation (Horgan et al. 2015)	VN1, VN3	PG, VN4	VN4, ID		
	Res. var.	Varieties have been developed using marker-assisted selection to pyramid genes for resistance to rice blast, bacterial blight (BB), and BPH (Ji et al. 2016, Korinsak et al. 2016)	PG				

^a^PRACTICED = is widely used in the country; RESEARCH = at research state only; READY=IPM alternative available for immediate implementation; ^b^frequently listed roadblocks, preventing technology diffusion: ENVI = it would not be effective under the environmental conditions of the country; ECON = deemed too expensive—so it is not widely adopted; ID (Indonesia), PG (Papua New Guinea), VN1, VN2,-VN3, VN4 (Vietnam)

Attract and kill strategies are the most commonly reported alternative pest control method, and attractants are regularly combined with insecticides or with entomopathogenic fungi (Vernon et al. 2016; Brandl et al. 2017). Besides attract and kill measures, non-systemic insecticides, synergists, surfactants, anti-resistance strategies and reduced dose applications are all described. For certain pesticidal products, targeted and well-timed foliar sprays can present significant advantages over unguided “blanket” drench applications or IPM-incompatible seed dressings (Kumar et al. 2012). Insecticide resistance development can also be reversed by entirely suspending insecticide use over specific time periods (Yang et al. 2014).

### Decision support systems

Decision support systems (DSS), including monitoring systems, action thresholds and predictive population models, are important pillars of IPM in all pest × crop systems. For western corn rootworm and wireworms, DSS are commonly put in practice (Fig. 1), with monitoring tools and predictive models used for wireworm control in the USA, Germany, Italy, Slovenia, and Hungary (Table 6). Similarly, for western corn rootworm, pheromone traps and yellow sticky cards are used for monitoring in nearly all countries though models are used to a lesser degree than for wireworms (Table 7). For bird cherry-oat aphid, forecasting models—based upon sowing time—are either in practice or “implementation ready” in Hungary, USA, Italy, and Spain (Table 3 and 4). For brown planthopper, intervention thresholds and population models are only occasionally put in practice—despite the prime importance of this pest in Asia’s rice crop (Table 9). Networks of light traps have been established in several Asian countries including China, Japan, Korea, and Indonesia (Horgan, personal observation); however, though these traps have helped characterize brown planthopper migration and assess the effect of meteorological parameters on migration patterns, they are not routinely used as early warning systems. In Vietnam, simple light traps have been employed at local scales to determine peak brown planthopper populations after which farmers can plant their rice crops (escape strategy: (Horgan 2018). Most of DSS tools or pheromone lures are not adapted to field conditions (40%), and the bulk of field-level records originate from Europe (40%, Table 1).

The overall aim of DSS development is to predict a pest’s population dynamics and to identify suitable intervention strategies (and their timing) based upon existing economic threshold levels. Modeling rhizosphere interactions can also help to assess the risk of soil pest attack (Johnson et al. 2016). For wireworms, a range of abiotic factors (e.g., altitude, precipitation, temperature, pH, organic matter content) can be incorporated as predictive variables (Jung et al. 2014; Staudacher et al. 2013; Hermann et al. 2013; Furlan et al. 2017a, 2017b). For aphid pests, abiotic factors, presence, and abundance of natural enemies and aphid identification modules are built into DSS models such as GETLAUS, CEAS, and APHIDSim (Gosselke et al. 2001; Piyaratne et al. 2013; Rossing et al. 1994; Gonzalez-Andujar et al. 1993; Kwon and Kim 2017). Aside from theoretical models (Wu et al. 2014; Fabre et al. 2010; Ma et al. 2001; Xian et al. 2007; Giarola et al. 2006), spatially explicit models can account for landscape composition and configuration, or for other factors such as wind speed and wind direction (Parry et al. 2006). Thresholds have been defined for wireworms (Furlan 2014), brown planthopper (Zheng et al. 2007) and for different cotton pests, including *A. gossypii* (Silvie et al. 2013; Sequeira and Naranjo 2008). Data input for DSS are regularly collected through field-level monitoring: WCR and wireworms can be monitored using sticky traps, pheromone traps or bait traps (Sufyan et al. 2011; Vuts et al. 2014; Benefer et al. 2012; Parker 1994, 1996; van Herk and Vernon 2013; Tóth et al. 2002; Tóth et al. 2007; Vuts et al. 2012; Tóth 2013; Tóth et al. 2015; Furlan et al. 2017a, 2017b; Bažok et al. 2011; Kos et al. 2014). In Northern Italy (Emilia-Romagna region) a monitoring net for two wireworm species has been employed in 2017 and 2018 using about 1100 pheromone traps per year, providing provisional threshold to alert farmers on infestation risk. For other pests, yellow pan trapping, sweep-net sampling or other kind of population assessments can be used.

### IPM approach

Although the principles of IPM are universally applicable, certain environmental and socio-economic factors can hamper IPM adoption (Vasileiadis et al. 2011). IPM entails the exhaustive use of non-insecticidal approaches (i.e., cultural, mechanical, phytosanitary practices) to prevent herbivores from reaching damaging population densities, draws on biological control as both a preventative and curative tactic, pursues the integrated use of mutually compatible technologies, and treats synthetic pesticides as a measure of “last resort” (Pedigo 1989). Prophylactic applications of systemic insecticides—e.g., as seed dressings or dips at the onset of the cropping season—are in direct conflict with this IPM concept, and have no room in IPM-managed systems. For bird cherry-oat aphid, the following measures do fit under the IPM umbrella: biological control, targeted chemical control—preferably with compatible products—as guided by decision rules and threshold levels, plant resistance and certain farming practices such as delayed sawing or nutrition management (Dedryver et al. 2010). For *A. gossypii*, mandatory dates for planting and harvest, post-harvest sanitation, and establishment of host-free periods along with—minimal—tactical use of insecticides can aid the recovery of cotton agro-ecosystems and concurrently lower pest pressure (Ellsworth and Martinez-Carrillo 2001; Naranjo 2001). Also, the establishment of groundcover, intercropping or trap crops help conserve resident natural enemy communities and prevent build-up of pest populations (Deguine et al. 2008). For *B. tabaci*, the IPM “pyramid” is composed of three main components: regular pest sampling, preventative measures, and a minimal, scientifically guided use of insecticides—prioritizing compatible insect growth regulators, IGRs (Ellsworth and Martinez-Carrillo 2001). Though genetically engineered crops are compatible with biological control (Romeis et al. 2018), large-scale, genetically uniform plantings of GM cotton can disproportionately favor whitefly pests. In those systems, measures can still be adopted to conserve arthropod natural enemies (Deguine et al. 2008). For aphid control, integrated weed and insect management strategies can reduce application costs without sacrificing the efficacy of either strategy, though full advantage needs to be taken of non-chemical measures (Ma et al. 2016). In rice systems, non-chemical technologies are well-advanced for insect pest management (Hong-xing et al. 2017). For rice brown planthopper, in addition to host plant resistance, adequate nutrient or irrigation management and conservation biological control (e.g.,(Gurr et al. 2016, Hemerik et al. 2018), particular fungicides interfere with pest development and can be included in IPM packages (Nanthakumar et al. 2012; Shentu et al. 2016). For each of the above pests, IPM packages are at different stages of development—ranging from scientific evaluation, farm-level validation and adaptation, to grower adoption. In Europe, complete and economically viable IPM packages are available for the management of wireworms and western corn rootworm in maize.

### Risk assessment and IPM “readiness” for selected crop × pest systems

Overall, pest risk levels were rated low to medium (i.e., brown planthopper) for all target pests, independent of the use of neonicotinoids (Fig. 2). Soil/seed treatments and foliar use of neonicotinoids varied among crop × pest systems and regions (Fig. 2), with nearly 100% of rice fields routinely treated against brown planthopper. Even so, 25–75% of those fields could be managed with alternative tools. Extent of field application with neonicotinoids was lowest for winter wheat (i.e., to control *R. padi*), and this likely can be entirely replaced with alternative tools (Fig. 3). In the following section, results from the expert evaluation and associated risk assessment are presented according to pest species.

### Bird cherry-oat aphid in winter wheat

Overall, pest status—and economic importance—of *R. padi* in European winter wheat is low to very low (0–25%) (Table 10, Fig. 2), largely because of a complex of effective resident natural enemies that colonize fields at the onset of the cropping season. In Slovenia, pyrethroid applications against the cereal leaf beetle *Oulema melanopus* (L.) can indirectly control *R. padi*. The pest is of local concern in certain areas, where foliar sprays are used for its control. In none of the European countries, experts voiced a need to use neonicotinoids as either seed or foliar applications (Table 10, Fig. 2). Yet, the current extent of reliance upon these products varied greatly between countries. In Spain, growers tend to resort to neonicotinoids to prevent barley yellow dwarf virus (BYDV) infection, though this is not regularly warranted: most Spanish farmers alter the sowing date to reduce aphid infestation and thus minimize the risk of virus attack. In Italy, 50-75% of fields rely upon CBC, and aphid infestations on organic farms are significantly lower than in those practicing IPM. In Slovenia, 25–50% of fields are managed through alternative tools—including those that rely upon IPM to manage *O. melanopus*. On the other hand, in the Western USA, 25–50% of wheat fields are planted with neonicotinoid-coated seeds, though the economic rationale for this high level of usage may be entirely lacking. Overall, biological control (including CBC), landscape-level management, farming systems adaptation (i.e., crop rotation, cultural practices), DSS, host plant resistance, and innovative pesticide application are available *R. padi* management tactics yet are not widely known or exist solely at the research stage. Most alternatives are either practiced or ready for implementation in Italy, Hungary, and Spain: CBC and landscape-level interventions are in practice in Italy and Spain. Locally, diverse and abundant arthropod natural enemies and entomopathogenic fungi exist in or near wheat fields and contribute to aphid biological control. In the Carpathian Basin, farmers recognize that an optimized plant density and nitrogen supply are cost-effective measures for *R. padi* control. Also, intercropping with oilseed rape, garlic or less susceptible varieties are interesting alternatives that are ready for implementation. In the Western USA, growers do alter sowing dates, while nutrition management tactics are still at the research stage. DSS such as the Cereal Aphid Expert System (CAES) are practiced in Italy and ready for deployment in Spain. Lastly, organic farming and full-fledged IPM packages are practiced in Italy and Slovenia.

**Table 10:** Actual use and potential reduction of neonicotinoids in arable crops and major alternative tools in practice (based on the expert consultation)

Combination	COUNTRY	REGION	IPM	High pest risk (% of crop land)	ACTUAL USE (% of crop land)	TARGET (% treated cultivated land)	IPM tools in practice
Maize/soil pests	Italy	North	a			< 5%	Crop rotation, monitoring, agronomic strategies such as tillage prior to sowing, attract and kill, biocidal plants, pheromone traps, DSS
	Italy	Rest of Italy	a		0–25%	< 5%	
	Hungary		a	0–25%	0–25%	5%	
	Poland		a	0–25%	0–25%	0%	
	Croatia		a	0–25%	0–25%	25%	
	Slovenia		a	25–50%	50–75%	25%	
	Germany		a	0–25%	0–25%	0%	
	Spain		a	0–25%	75–100%	10%	
	USA	Western US	a	0–25%	75–100%	< 25%	
	USA	IOWA, Indiana	a		75–100%	< 5%	
Winter wheat/soil pests.	Italy	North	a	0–25%	0–25%	0%	Crop rotation, monitoring, agronomic strategies such as tillage prior to sowing, attract and kill, biocidal plants, pheromone traps, DSS
	Italy	Rest of Italy	a	0–25%	0–25%	0%	
	Hungary		a	0–25%	0–25%	0%	
	Croatia		a	0–25%	0–25%	0%	
	Slovenia		a	0–25%	0–25%	0%	
	Spain		a	0–25%	0–25%	0%	
	Germany		a	0–25%	0–25%	0%	
	USA	Western US	a	50–75%	75–100%	0%	
	USA	IOWA, Indiana	b	0–25%		< 5%	
Winter wheat/aphid	Hungary		a	0–25%	0–25%	0%	Conservation biological control, landscape management, nutrition management, plant density and sowing date, DSS
	Croatia		a	0–25%	0–25%	0%	
	Italy		a	0–25%	0–25%	0%	
	Slovenia		a	0–25%	0–25%	25%	
	Spain		a	0–25%	0–25%	0%	
	USA	Western US	a	0–25%	25–50%	0%	
Rice/plant hopper	Vietnam	North	bc	25–50%	50–75%	25–50%	water management, nutrition management, resistant varieties, 3 reductions program, 5 reductions program
	Vietnam	South	ac	0–25%	0–25%	0%	
	Vietnam	South (Mekong Delta)	bc	25–50%	75–100%	25–50%	
	China		c	25–50%	75–100%	25–50%	
	Philippines		c	25–50%		25–50%	

(a) Ready—immediate implementation; (b) to be adapted; (c) to be set up

None of the experts evaluated bird cherry-oat aphid as a high-risk pest (Table 10, Fig. 2), possibly due to the low degree of usage of non-selective insecticides, an enhanced adoption of field-level diversification tactics, and a resulting increased impact of locally occurring natural enemies. In Europe, CBC can be the primary alternative to neonicotinoids, while nutrition management, host plant resistance and DSS carry ample potential for further development, fine-tuning, and promotion (Gosselke et al. 2001; Gonzalez-Andujar et al. 1993; Day et al. 2015). In the USA, *R. padi* was equally perceived to be a low-risk pest, yet 25–50% of current winter wheat acreage is annually sown with neonicotinoid-coated seeds. For a low-risk pest, such high levels of neonicotinoid use seem unwarranted.

### Wireworm in maize and winter wheat

Wireworms were historically regarded as limiting pests of several cultivated crops in Europe. However, our expert assessment reveals that the perceived risk of wireworms is generally low, except for Croatia (25–50%) and the western USA (25–50%). A long-term risk assessment is available for wireworm in Europe. Furlan et al. (2017a) showed how certain parameters (e.g. grassland in rotation or in the vicinity of the field) greatly increase the risk of crop damage from wireworms in maize. The probability of economic damage was less than 4% (as studied over a 29-year period) in Italy, and these patterns equally hold for other EU countries. Overall, plant damage was low or even negligible in most cases (> 90% had less than 5% wireworm plant damage). In (few) cases with > 15% plant damage, maize yield did not differ between untreated plots and those were soil insecticide was used. The decision to treat crops with soil insecticides (including neonicotinoid seed coating) is based exclusively on wireworm risk assessment, considering that no chemical treatments at maize sowing are needed to control black cutworm, western corn rootworm, and other minor soil pests (Kaster and Showers 1984; Furlan and Kreutzweiser 2015). Some local exceptions may occur to address e.g. *Tanymecus dilaticollis* Gyll. damage in some areas of Romania (Saringer and Takács 1994); thus local adaptations of IPM strategies need to be considered. Even in areas where wireworms are ranked as a key pest, their impact on crop yields tends to be low and possibly of minor economic importance (Table 10, Fig. 2), except for Pennsylvania (USA), where wireworm damage—though often confined to certain areas of a field—can cause stand losses up to 75–80%. Wireworm damage is patchily distributed, usually remains undetected and infrequently exceeds 15% of a standing crop. It can be severe in some cases—reaching high infestation levels. In Europe, no soil insecticides are used in winter wheat and no significant wireworm damage is recorded, though maize fields are regularly treated with soil insecticides, including neonicotinoids (Table 10, Fig. 2). In the Western USA however, virtually 100% of maize and wheat fields are treated with neonicotinoid soil-insecticides. As resident wireworm populations are likely to be of minor economic importance, there is ample room for implementation of non-chemical alternatives (Table 10, Fig. 2). For example, incorporating barley and oats into crop rotations can reduce wireworm attack (Milosavljević et al. 2019). In the US Midwest, near-universal applications of soil insecticides are directed against western corn rootworm, while negligible wireworm damage is recorded in untreated plots. In Europe, wireworm management differs greatly between countries and production areas. In Slovenia, 40–65% of maize growers use soil insecticides or seed treatments, and similar patterns are reported for Italy. Although no specific measures are used against wireworms, crop rotation, tillage, non-neonicotinoid insecticides and the planting of biocidal plants are widely practiced by local growers. Slovenian livestock producers are affected by wireworms when including meadows in rotation schemes and regularly revert to neonicotinoids. Yet, most producers do not conduct pest monitoring, and thus remain uniformed whether local wireworm populations exceed economic thresholds and cause economically significant losses. In Spain, insecticidal soil treatments are regularly used in a prophylactic manner and may be largely superfluous.

Our expert evaluation revealed how numerous tools are either practiced or “implementation ready” (Tables 5 and 6). Biological control, e.g., entomopathogenic fungi *Beauveria bassiana* and *Metarhizium anisopliae*, are used in Germany and ready for use in Italy and Slovenia. In Hungary, environmental conditions (i.e., drought) may limit the use of nematodes but could favor field application of *Metarhizium* spp. In the USA, biological control options are mainly at a research stage. Crop rotation is commonly practiced in all countries. In Hungary, the use of *Phacelia tanacetifolia* as green manure causes high levels of pest mortality, while the repellent effect of CaCN_2_ is recognized in Italy, but not yet practiced. In Germany, the latter alternative is practiced, but does require a careful timing of application. Lastly, tillage is widely adopted as a management strategy in Hungary and Spain.

Monitoring tools and DSS in general are practiced in the USA but only used to a limited extent in Europe (e.g., Hungary, Slovenia). In Italy’s Emilia-Romagna region, a monitoring network with pheromone traps has been deployed to forecast area-wide risk of wireworm attack: when a 700–1000 captures/trap threshold is reached for particular wireworm species, larval sampling is suggested to further guide management actions. Also, once the above threshold is exceeded, the use of soil treatments is restricted to max. 50% of field area. In Germany, simple traps are used and abiotic risk factors are taken into account for wireworm management. Attract and kill and alternative insecticides are either unknown or not applicable for most of the countries given existing restrictions on the use of fipronil, bifenthrin and lindane. In Germany, attract and kill is used with a different product (ATTRACAP), while chlorpyrifos is used in Spain for soil treatment—though concerns exist over its potential environmental impacts.

### Western corn rootworm in maize

Overall, the risk of western corn rootworm damage was rated low (except Slovenia). However, western corn rootworm risk is inflated in systems with continuous mono-culture planting, as compared to those where crop rotation is used. Especially dairy farmers appear to be reluctant to adopt crop rotation given their often-exclusive reliance upon maize as animal feed, and regularly suffer western corn rootworm damage of up to 50%. Yet, there are ample rotation options that can improve both milk quality and farm profit (Furlan et al. 2018). Across Europe, neonicotinoids are used in less than 25% of maize area (Table 10, Fig. 2). In contrast, in the USA, most maize fields are treated with soil-applied neonicotinoids (Table 10, Fig. 2) and 25–50% of untreated fields are effectively managed with alternative tools. Hence, there is ample potential for these tools to be used over substantially greater areas.

Most alternatives are exclusively at the research stage or not widely known (Table 7), except for crop rotation and pheromone trapping for monitoring purposes. In Hungary, models to guide crop rotation are used. In the USA, Diabrotica-resistant GM corn hybrids are used along with planting refuges. Lastly, nematode biological control is ready for use in Germany and GM tactics wait to be deployed in Hungary. Our expert evaluation yielded few western corn rootworm management tools. In Slovenia, DSS are available that use degree-day models to predict adult emergence while pheromone-based mating disruption and nematode application is at the research stage. In Germany, laboratory research is ongoing to refine attract and kill using CO_2_-release capsules and *Metarhizium* fungi. Experts regularly list environmental and economic impediments to the further adoption and up-scaling of biological control.

Despite low rate of seed or soil treatments (except for Slovenia), the perceived western corn rootworm risk across Europe is still low. In the USA, there is ample potential to reduce reliance upon neonicotinoids, as numerous alternatives are already in practice: crop rotation, *Bt* corn, monitoring traps, refuges along with Diabrotica-resistant hybrids. The main driver of western corn rootworm attacks is continuous corn planting, and system-level changes—e.g., adoption of crop rotation—can thus drastically lower pest issues. Biological control with *Bt*, nematodes, or entomopathogenic fungi and biopesticides is equally effective: *Azadirachta indica* A. Juss. (L.) fruit and leaves have insecticidal effects, while *Gliricidia sepium* and turmeric act as repellents. Attract and kill options can either use natural attractants (e.g., powdered roots of buffalo gourd, corn seedling volatiles, CO_2_, extracts of germinating corn) or synthetic volatiles to attract larvae. Lastly, pheromone-based monitoring can feed DSS and guide farmers’ management decisions. In conclusion, multiple alternatives and IPM technology packages are well-tailored to maize production systems globally, and can simultaneously resolve wireworm and western corn rootworm issues. European maize and wheat has historically been grown in a profitable fashion without any chemical insecticides. If today’s growers can steer clear of continuous maize cropping, they can side-step wireworm issues and avoid financial expenditures for insecticide usage.

### Brown planthopper in rice

Largely considered a minor rice pest until the mid-1960s, *Nilaparvata lugens* has assumed the status of destructive pest due to “green revolution” style crop intensification (Pathak and Dhaliwal 1981; Heinrichs and Mochida 1984). Though sharp reductions in pesticide use restored natural enemy communities and resolved *N. lugens* pest issues during the 1980s and 1990s, (neonicotinoid) insecticides are once again increasingly used, leading to major insecticide resistance issues and triggering *N. lugens* outbreaks over extensive areas in tropical Asia (Bottrell and Schoenly 2012). In general, *N. lugens* outbreaks are indicative of crop mismanagement, insecticide abuse and unsustainable rice intensification (Sogawa et al. 2009). In only a few areas, the risk of *N. lugens* is considered zero e.g., in a high percentage of fields in southern Vietnam. Adoption levels of alternatives greatly vary between sites and individual countries.

Overall, the pest status of brown planthopper was considered medium (25–50%), and its economic damage was rated as low to medium by experts. Yet, neonicotinoid granules are used on 50–75% of the rice area in Vietnam’s Mekong Delta and on 25–50% fields in northern parts of this country (Table 10, Fig. 2). In China, no granular neonicotinoids are used, and 50–75% of untreated fields are managed using alternatives (Table 10). However, both Chinese and northern Vietnamese rice growers adopt foliar sprays of neonicotinoids in 75–100% of fields—though alternatives could readily be used over much of this area (Fig. 2). The high use of insecticide in northern Vietnam and China is associated with high adoption of hybrid rice varieties (> 70% of rice production area), many of which are hyper-susceptible to the white planthopper, *Sogatella furcifera*, and susceptible to brown planthopper (Horgan and Crisol 2013). In recent years, much research attention has been placed on developing improved hybrid varieties with resistance to both planthopper species (Horgan 2018). In southern Vietnam, large numbers of fields are treated with alternatives as promoted through national programs such as Three Reductions Three Gains (3R3G—reduce seeds, fertilizer, pesticides and gain yield, crop output, and net income) or the “1 Must Do 5 Reductions” (1M5R), which entails using certified seed while pursuing reductions in seed, fertilizer, chemical pesticide inputs, water use, and post-harvest losses.

Alternative tools are practiced in several key rice-producing regions: fertility management (i.e., silicon addition, balanced nitrogen inputs) and resistant varieties are adopted in Vietnam and Indonesia. On the other hand, adapted potassium fertilization is ready for implementation in some countries. Also, IPM packages consisting of appropriate water management (i.e., avoidance of water-stress), insecticide reduction and host plant resistance are adopted in Vietnam. In both Vietnam and Indonesia, the planting of varieties with brown planthopper resistance genes has been hindered by the widespread and rapid adaptation of planthoppers to resistant varieties; however, some success was achieved in Indonesia with the local variety Inapari 13. Landscape-level diversification is practiced in Indonesia and in southern Vietnam, and is ready for implementation in the Philippines. However, although the effects of diversification on planthoppers have been relatively well studied, their impact on multi-species pest complexes has been difficult to anticipate and diversification recommendations thus need to be fine-tuned and locally adapted. Numerous additional tools were considered to be “implementation ready”: biopesticides for example are ready to use in Vietnam. In the Philippines, many alternatives have received research attention, but their practical application (or field-level evaluation and adaptation) is lagging.

Experiences in southern Vietnam and in Indonesia show that holistic, systems-level interventions that combine good agronomy (including the incorporation of organic matter and animal manure), synchronous planting, host plant resistance, and biological control (e.g., 3R3G, 1M5R, or the application of plant growth promoting rhizobacteria), could successfully lower—or even completely eliminate—synthetic insecticides. These kinds of approaches urgently wait to be transferred to other areas and adapted to meet local growers’ needs, conditions, and farming contexts.

## Discussion

This study offers a synthetic review of the extent of usage of neonicotinoid insecticides in four globally important arable cropping systems (i.e., wheat, maize, rice, and cotton), and provides a systematic listing of non-chemical alternatives to replace these products in each system. Our work shows that neonicotinoid use is highest in rice against brown planthopper and in maize against soil-borne pests, and lowest in winter wheat against bird cherry-oat aphid and wireworms though only based on European data. For each of the crop × pest systems, myriad well-tested, cost-effective alternatives are available to swiftly transition away from neonicotinoids. As insect herbivores generally pose low risk in European cereal systems, we do not anticipate notable increases in crop damage (or declines in farm-level revenue) following the continent-wide ban on various popular neonicotinoids. Instead, current EU-wide restrictions on the use of imidacloprid, clothianidin, and thiamethoxam will help restore on-farm biodiversity, strengthen ecosystem services, and enhance in-field biological control. Furthermore, multiple alternatives are available at differing stages of readiness, several of which have been validated, adapted, and successfully used by farmers. Alternatives are locally adopted by non-negligible numbers of farmers in winter wheat to control bird cherry-oat aphid (in Hungary and in Spain), yet farmers wait to adopt alternatives for wireworm management in most countries (Italy, Croatia, Germany, USA). Similar to cereal-based systems, numerous IPM alternatives are available and validated for maize systems, but their farm-level adaptation has not yet fully been realized, especially outside Europe. This may be explained by the higher gross margin of maize compared to cereals, or to the different land-use patterns and agro-landscape structure in Europe as compared to the rest of the word. Overall, we can confidently say that farmers who adopt non-chemical alternatives in small cereals and maize systems are likely to increase profitability of their operations, protect the environment while securing a steady output of safe, nutrient-rich farm produce.

For each of the target pests, despite the current over-reliance on neonicotinoids, promising trends can be observed in all arable crop systems. In the USA and Spain, current coverage of neonicotinoid-treated cereal crops is very high, and there may be considerable potential for reduction. For maize and winter wheat, IPM packages and “regenerative” farming schemes have been devised and field-tested, under which crop yields are sustained and farm profit can even be doubled (e.g., (LaCanne and Lundgren 2018)). For *A. gossypii* and *B. tabaci*, though alternatives are well-described in the literature, research findings urgently need to be translated into practice. In certain systems, e.g., Arizona cotton, IPM packages consisting of altered planting dates, sanitary measures, and host-free periods permit a drastic reduction in insecticide use while maximizing field-level abundance and pest suppression potential of natural enemies (e.g., Ellsworth and Martinez-Carrillo (2001); Naranjo (2001)), and these experiences can readily be transferred to other production regions, e.g., in China, Pakistan, Egypt, or West Africa. Also, the well-developed biological control programs for pests such as *Bemisia tabaci* in greenhouse settings can help feed the design of CBC schemes (and possibly augmentative biological control interventions) in open-field crops. Our survey reveals comparatively high levels of neonicotinoid use in rice production, and low degrees of adoption of alternatives (except for areas in Vietnam, where rice growers have embraced 3R3G or 1M5R). More research is needed to develop full-fledged IPM packages that need to be validated by farmer groups. For brown planthopper in rice, one can now build upon initial successes with these 3R3G or 1M5R, and pursue a further incorporation of semio-chemicals, ecological engineering tactics and agronomic measures to achieve further reductions in insecticide use; however, past successes have been achieved through dedicated attention and due investment in communication strategies and campaign-type implementation. Evidence suggests that once funding for such communication campaigns declines or the campaigns otherwise cease, insecticide use will likely increase (Horgan 2018).

For all crops, organic farming practices can equally restore or bolster ecosystem services such as biological control and help suppress pest populations, though their efficacy is likely pest-dependent (Muneret et al. 2018). In rice, participatory farmer training programs—eventually complemented with mass-media communication campaigns (including farmer-to-farmer video)—can help validate and adapt non-chemical alternatives to local farming contexts and rice production typologies (e.g., upland, low-land paddy). To facilitate these transitions, we introduce some guiding concepts and illuminate examples of successful agricultural extension (and transformation) programs in the section below.

### From theory to practice: facilitating the diffusion of alternatives

Our work reveals how several nuclei of farmers worldwide have successfully transitioned away from neonicotinoid insecticides, and instead employ non-chemical management alternatives. Opportunities exist to accelerate this process, engage more growers in regenerative styles of farming and ultimately reach a “tipping point” towards ecological intensification (e.g. (Tittonell 2014, Bommarco et al. 2013, Pretty et al. 2018). In order to enable this transition, a sound understanding is required of the various factors that shape farmers’ technology adoption and the relative contribution of, e.g., cultural, social, economic, climatic, agronomic and in-field ecological processes. Farmer decision-making is complex, and a “systems-level” perspective is essential to fully appreciate why growers in particular localities refrain from using, e.g., biological control while continuing to rely upon (insecticide-based) approaches despite their—often—questionable efficacy, cost-effectiveness, and environmental profile. To successfully upscale alternatives, a focus on “innovation systems” instead of technical particularities of individual technologies is required (Schut et al. 2014), and an integration of individual practices under an IPM umbrella is a must (Stenberg 2017). Using Rogers’ (1962) “Diffusion of innovations” framework, Wyckhuys et al. (2018) identified five key “roadblocks” for a broader adoption of biological control. We adopt this same framework to examine current adoption patterns of neonicotinoid alternatives, and list concrete opportunities to remediate certain “roadblocks” for individual farming contexts and crop × pest systems.

#### Availability of sufficient knowledge on neonicotinoid alternatives

Diagnosing the “readiness” status of alternative technologies in 7 pest × crop complexes, our works reveals an immense disparity in the local availability of alternatives (and supporting ecological knowledge) between cropping systems, IPM categories and geographies. For example, while 7 different management alternatives are “under research” for brown planthopper in Papua New Guinea, there is only one biological control option “ready for implementation” and none in practice. Earlier work has revealed an overall absence of CBC options for several of the world’s crops and accentuated how multiple insecticide-importing nations have limited or no alternative technologies on offer (Wyckhuys et al. 2013), with only a few commercially available natural enemies in the tropics (van Lenteren 2012). As local absence of alternatives effectively impedes their field-level adoption, our work calls for an acceleration of applied research in rice and for a (farm-level, participatory) technology validation in rice and maize. As a next step, locally validated technologies can be shared with farmers and the general public through, e.g., (mass-media) extension campaigns, “innovation” platforms for knowledge co-creation and sharing, farmer-to-farmer video channels, or online public media (Van Mele et al. 2009; Pretty et al. 2018; Wyckhuys et al. 2019c).

#### Divergent interests and priorities of farmers

In their daily chores, farmers have to find a delicate balancing act, diverting their attention, time and (often scarce) resources to address multiple concerns. Unpredictable weather patterns, inadequate plant nutrition, crop failure, shifts in availability (or pricing) of inputs and supplies, and fluctuations in demand for harvested produce are all issues on farmers’ minds, and shape farming decisions. Insect pests indeed can constrain crop production and have been shown to reduce yields by 10–16% worldwide (Oerke 2006), and farmers thus rightly worry about an eventual occurrence of pest outbreaks. Also, given their busy schedules, risk-averse farmers with sufficient financial resources regularly favor practices that circumvent laborious monitoring and require little thought (e.g., calendar-based sprays or “convenience” application modes), so-called “lazy-man tactics” (i.e., insecticide seed coating) and other preventative measures. Also, farmers’ actions are often guided by their beliefs and perceptions—instead of by actual pest numbers, pest-induced crop loss or the real financial implications of taking pest control action (Heong et al. 2002; Mourtzinis et al. 2019). Given that our expert panel ranked all target pests as “low- to intermediate-risk” and that many pest problems are secondary (i.e., triggered by farmers’ own insecticide use), it is clear that those perceptions—and associated actions—are radically misguided. For example, in cotton production in the San Joaquin Valley (California), farmers who opt for “preventative” early-season insecticide sprays suffered from secondary pest problems and spent an additional $15/ha to resolve those, once again with synthetic insecticides (Gross and Rosenheim 2011). In Nicaraguan cabbage production, farmers who refrained from insecticide use ran substantially higher profits than those who did not (Bommarco et al. 2011); similar findings have been made for Philippine and Indonesian rice systems. Well-conceptualized and concerted efforts to rectify farmers’ perceptions (and related risk-averse behavior) can thus prevent induced pest issues while greatly benefiting farmers’ pockets.

#### Weak (agro-ecological) knowledge base

When interviewing farmers in the 1990s, anthropologists were regularly told “nothing kills insects, except for insecticides” (Wyckhuys et al. 2019a, 2019c). Though one might expect shallow ecological knowledge among illiterate, unschooled or resource-poor growers in the developing world, similar patterns—rather surprisingly—have been recorded among contemporary farmers in Western Europe (Zhang et al. 2018). Aside for honeybee pollinators and ladybugs, human beings somehow face supreme difficulties to recognize or enumerate beneficial invertebrates irrespective of their multi-billion dollar contribution to pest management (Losey and Vaughan 2006). For example, overall knowledge of insects among USA college students is restricted to a mere 13 species (Bixler 2017). Switzerland and Japan—countries that top the ranks globally in terms of general education—reported similar patterns (Breuer et al. 2015; Hosaka et al. 2017). In Canada, a national phone survey recorded a positive attitude towards biological control, but also commended intensifying tailored outreach and education (McNeil et al. 2010). In addition to a general disinterest or even fear towards invertebrate natural enemies (including spiders), locally held beliefs can preclude the on-farm trialing and adoption of non-pesticidal alternatives such as biological control (e.g., (Winarto 2004)). Many of the alternatives outlined in this paper are knowledge-intensive, i.e., require a fair degree of specialized (agro-ecological) knowledge on behalf of farmers to secure their successful on-farm adoption. Scientists often assume that farmers do possess the necessary knowledge base to successfully implement IPM; yet this assumption is false. In fact, the vast majority of farmers (and the general public) has no understanding whatsoever of insect-killing fungi or viruses, minute endo-parasitoids or predatory mites (Wyckhuys et al. 2019a). Hence, thoughtfully crafted communication initiatives are required to build or strengthen farmers’ ecological knowledge, provide workable alternatives and steer their decision-making away from costly and environmentally damaging insecticides.

#### Perceived attributes of alternatives

Several elements inherent to pest management—and perceived by individual farmers in different ways—can either accelerate or impede the uptake and subsequent diffusion of non-chemical alternatives. Five technology attributes in particular constrain the adoption of alternatives such as biological control (Wyckhuys et al. 2019c; Wyckhuys et al. 2018): (i) relative advantage, (ii) compatibility, (iii) complexity, (iv) traceability, and (v) observability. More specifically, (i) USA walnut and pear growers praise the low (financial, human health) cost and environmentally friendly profile of biological control, though often question its advantage in terms of effectiveness (Goldberger and Lehrer 2016). Despite major geographical and temporal variability in cost-effectiveness and yield benefit (Tooker et al. 2017) and inconsistent benefits for farm-level profitability (LaCanne and Lundgren 2018)), neonicotinoids seemingly have other comparative advantages that explain their present-day use on tens of millions of hectares worldwide. (ii) As the efficacy of biological control is often context-dependent, complementary on-farm and landscape-level CBC actions can be taken to bolster its success rates (Shields et al. 2019). Also, certain alternatives are not compatible with (conventional) farm management schemes, e.g., when there is zero weed tolerance on large-scale “manicured” farms (Marshall et al. 2003) or when unguided insecticide applications remain in use (e.g., (Fogel et al. 2013). (iii) A third possible impediment is the (perceived) complexity of alternatives such as CBC floral strips (Gurr et al. 2016), beetle banks or DSS, as compared to neonicotinoid seed coatings—which are readily applied at the time of seed drilling. Certain technologies—such as the non-use of insecticides (Goldberger and Lehrer 2016)—are far less complex, and could yield satisfactory results when coupled with supporting CBC measures and promoted through thoughtful, targeted messaging. (iv) Neonicotinoid-based technologies regularly score high in terms of trialability, as it is easier for a farmer to test the efficacy of seed coatings in a field corner than to effectively trial, e.g., habitat management interventions. In Asia’s rice-growing areas, insecticides are sold “over the counter” in small sachets—similar to fast-moving consumer goods such as candy bars, soap or shampoo, thus further encouraging their trial-adoption. To encourage farmers with the trialing of alternative technologies, one-time economic incentives could be considered (e.g., (Cullen et al. 2008). (v) Observability is a major impediment for technologies that rely upon the use of, e.g., small-sized predatory mites or endophagous parasitoids. On the other hand, farmers—across the globe—take joy in observing the “knock-down” effect of certain insecticides and are often satisfied when there is a total absence of insects—irrespective of them being pests or natural enemies—in crops established with neonicotinoid-coated seed. Hence, when developing and up-scaling non-chemical alternatives, it is important to thoroughly examine the above technology attributes (and the associated decision-making processes).

#### Perceived type of innovation-decision

Three types of innovation-decision (Rogers 1962), can be distinguished: (i) optional, (ii) collective, and (iii) authority innovation-decisions. All three types are relevant when promoting neonicotinoid alternatives and carry variable potential under different geographical, crop × pest or socio-cultural contexts. A fair share of management decisions in European or USA agriculture are directly made by individual farmers. This may be different for contract farming, where there might be both lucrative opportunities (Sullivan et al. 1999); for Guatemalan snow pea) and important roadblocks (Grossman 1999); for conventional plantation-style banana) to further ecologically centered IPM. The role of collective decision-making processes can best be exemplified by the voluntary enrollment in mutual funds and pest-insurance schemes by entire groups of Italian maize growers, collective efforts to pursue agricultural systems “redesign” (Pretty et al. 2018), or farmers’ organized supply of “quality produce” to supermarkets, e.g., in Vietnam (Moustier et al. 2010). The UN-sponsored “area-wide pest management (AW-IPM)” approach (Vreysen et al. 2007) possibly could be used as an operational framework to phase out neonicotinoid insecticides over large areas and collectively move toward implementation of alternatives. Under such schemes, authority decisions can equally help propel alternatives, as exemplified by Cuba’s 1990s conversion of a staggering 1 million ha to biological control under well-coordinated, state-sponsored programs (Nicholls et al. 2002). Several of the above *modus operandi* carry potential to deliver pest management alternatives for the arable crops covered in this paper and can help ensure lasting (if not transformative) change at scale.

## Conclusion

Our work reiterates how (neonicotinoid) insecticides are not necessarily employed to resolve economically important pest issues, but instead often constitute superfluous cost components in farming operations. Their unguided use can further trigger pest resurgence, degrade ecological resilience of agro-ecosystems and compromise long-term farm profitability. Also, their prophylactic application (e.g., as seed coatings or stem dips) is in direct conflict with globally valid IPM concepts and contributes to biodiversity loss. Our expert panel and scientific literature review reveal how (a) in most systems, pest populations rarely exceed economic threshold levels and the recurrent broad-scale (often prophylactic) use of these products is unjustified; (b) effective IPM procedures and tools are available to immediately reduce or suspend (neonicotinoid) insecticide use; and (c) that such insecticide phase-out can help improve or sustain farm-level revenue streams. Our study identifies several effective alternatives to (neonicotinoid) insecticide use in most important arable crops in the world; some of these alternatives are ready to be used for all the crop × pest combinations. The first and most powerful alternative is just the concrete implementation of the IPM principles: low cost pest risk assessment with complementary limited in field evaluation to identify fields that do not require pest control. For most crop × pest combinations, practical methods are available to identify fields where pest control is needed. Their field-level implementation can be facilitated by establishing an effective independent advisory system and by providing insurance tools that make farmers comfortable with IPM implementation. As to *Diabrotica*, rotation proved to be the most effective and sustainable alternative. Rotation schemes may be flexible: maize may be rotated at varying frequencies (even after several years), only when monitoring reveals that WCR population levels are increasing, as demonstrated in practice by Furlan et al. 2018. For most crop × pest combinations, landscape management increasing biodiversity proves to be a sound as it can bolster biological control. In rice, pest-resistant varieties can mitigate insecticide use against *Nilaparvata lugens*.

To facilitate the broad diffusion and farm-level implementation of IPM alternatives, it is necessary to pursue the following five steps: (1) effectively communicate low-cost, labor-saving IPM alternatives among a broad range of stakeholders, including farmers, to trigger farmer experimentation, induce innovation and stimulate technology diffusion (Furlan et al. 2017b); (2) set precise and verifiable targets for IPM implementation for each crop × pest system in the different geographies (e.g., annually diminishing maximum % of insecticide-treated cultivated land); (3) create or re-constitute an independent advisory system that provides objective guidance and scientifically underpinned information on local availability and efficacy of (non-chemical) alternatives; (4) support insurance approaches—e.g., mutual funds—to account for eventual agro-ecological upsets and uncertainties involved in IPM implementation; (5) carry out comprehensive, unbiased risk assessment and development of plant health strategies for those crop × pest systems that currently lack solid and effective IPM packages. By judiciously following these steps, deploying supportive policies and enabling an effective implementation of ecologically centered IPM, neonicotinoid insecticide use can be scaled down swiftly and substantially. Doing so will carry considerable benefits for the environment, farmers and society at large.

## Electronic supplementary material

ESM 1 (DOCX 1720 kb)
