# Supplementary material for: An update of the Worldwide Integrated Assessment (WIA) on systemic pesticides. Part 4: Alternatives in major cropping systems
Source: Environ Sci Pollut Res Int. 2020 Jun 4;27(24):29867–99. doi: 10.1007/s11356-020-09279-x (PMC7378116; doi:10.1007/s11356-020-09279-x)
Supplement: Supplementary file 1 — (DOCX 1720 kb) [file 11356_2020_9279_MOESM1_ESM.docx]

**Supplementary material**

Table 7. Pest management alternatives identified through the literature review for aphid (*Aphis gossypii*) in cotton

| Categories | Sub-categories | IPM alternatives identified |
| --- | --- | --- |
|  |  |  |
| Biolog. cont. | *Biopesticides* | Solvent extracts of *Chenopodium ficifolium* as botanical insecticide |
|  | *Biopesticides* | Sandalwood oil (*Santalum austrocaledonicum*) can be used for cotton aphid control |
|  | *Fungi* | *Lecanicillium longisporum* (Vertalec) is highly pathogenic against adult aphids with a lethal time (LT50) of 6.9 days |
|  | *Fungi* | Naturally-infected insects can be used as source of the fungal pathogen *Neozygites fresenii*, as done in commercial cotton fields in Arkansas |
|  | *Fungi* | An isolate of *Lecanicillium attenuatum* CS625 has high virulence against cotton aphid, upon treatment with fungal conidia or blastospores |
|  | *Fungi* | *Lecanicillium lecanii* 41185 showed the highest virulence for cotton aphid (and other aphid species) |
|  | *Fungi* | Conidia of *L. lecanii* and *B. bassiana*. reduced aphid reproduction and reproductive period, lowered settlement rates and reduced survival rates |
|  | *Co-infection* | Endophytes *Fusarium oxysporum* and *Rhizobium etli* affect aphid feeding behavior, with aphids preferring endophyte-free plantlets |
|  | *self-induced def.* | Volatiles from seedlings treated with the naturally-occurring plant defence elicitor cis-jasmone (CJ) were repellent |
|  | *Classical* | An Arkansas strain of the fungus, *Neozygites fresenii* was released for aphid control, using dried *N. fresenii*-infected aphid “cadavers” and ‘chamber inoculation’ the latter resulting in higher prevalence of fungal infection |
|  | *Landscape manag.* | Landscape diversity was negatively associated with ladybeetle density; presence of maize fields enhanced ladybeetle numbers while in-field applications of insecticides lowered ladybeetle density |
| Cultural or mechanical control | *Nutrition management* | Addition of potassium fertilizer reduced cotton aphid population density when nitrogen fertilizer was not used; aphid population density at seedling stage was suppressed by a proper combination of K and N fertilizer (K:N = 1:0.9 or 1:1.2 kg/ha). |
| DSS | *Threshold* | Threshold-based interventions for aphid pest control |
|  | *Model* | A simulation model predicting first (male) aphid occurrence based upon day length and temperature |
|  | *Model* | Aphid population dynamics are predicted based upon host plant type and climatic variables such as temperature and precipitation, and results can be used to guide biological control. |
|  | *Resistance detection* | Following the appearance of neonicotinoid-resistant clones in Japan, a multiplex polymerase chain reaction method was developed that targeted a point mutation (R81T) in the nicotinic acetylcholine receptor gene, thus enabling rapid diagnostics |
| Innov. pestic. and appl. reg. | *Formulation* | Lauric acid, a type of middle-chain fatty acid, kills aphids by suffocation and desiccation |
|  |  | expression level of Dicer-1 was significantly increased when adults fed on plant allelochemicals (tannic acid and gossypol) incorporated artificial diets. |

a PRACT=is widely used in the country; RESEAR= at research state only; READY=IPM alternative available for immediate implementation; b frequently listed roadblocks, preventing technology diffusion: ENVI= It would not be effective under the environmental conditions of the country; ECON= deemed too expensive - so it is not widely adopted

Table 8a Pest management alternatives identified through the literature review for silver-leaf whitefly (*Bemisia tabaci*) in cotton

| Categories | Sub-categories | IPM alternatives identified |
| --- | --- | --- |
|  |  |  |
| Other | *Herbicide* | Mixed application of the herbicide glyphosate and the insecticide acephate improved aphid control; a judicious integration of weed and insect management strategies possibly can reduce application costs without sacrificing the efficacy of either strategy |
| Biological control | *Classical* | The parasitoid *Eretmocerus mundus* has permanently established in California, in efforts to implement biological control against *B. tabac*i biotype “B” |
|  | *Fungi* | The fungus *Isaria fumosorosea* IF-1106 infects all sweet potato whitefly lifestages |
|  | *Fungi* | When using entomopathogenic fungi against *B. tabaci*, it is recommended to initiate treatments against early stages of the pest, target pest populations under moderate environmental conditions (e.g., spring or fall seasons), select crops amenable to multiple spray applications, and alternate fungal sprays with those of (incompatible) fungicides |
|  | *Fungi* | The fungi *Verticillium lecanii*, *Paecilomyces fumosoroseus* and *Beauveria bassiana* carry ample potential for use against *B. tabaci* |
|  | *Fungi* | The fungi *B. bassiana* and *I. fumosorosea* attained high virulence against nymphs, though prove ineffective against egg stages of *B. tabaci* |
|  | *Inter.fungi &predator* | The entomopathogenic fungi *Lecanicillium longisporum* and *L. muscarium* are disseminated by the predatory bug, *Orius laevigatus* thus boosting whitefly biological control |
|  | *Inter.fungi & parasitoid* | The entomopathogenic fungus *Lecanicillium muscarium* does not negatively affect the whitefly parasitoid *Eretmocerus sp.* |
|  | *Parasitoid* | In cage assays, the parasitoid *Amitus bennetti* inflicted 61% whitefly mortality on cotton |
|  | *Parasitoid* | High levels of whitefly biological control were attained using *Eretmocerus* parasitoid species from regions with similar climatic conditions |
|  | *Predator* | Augmentative releases of the ladybeetle *Delphastus catalinae* for whitefly biological control did not adversely affect population densities of indigenous parasitoids, suggesting an absence of strong antagonistic predator–parasitoid interactions. |
|  | *Parasitoid* | Different species of parasitoid - including *Encarsia bimaculata*, *Encarsia porteri* and *Eretmocerus hayati* attack *B. tabaci* and possibly can be incorporated in biological control programs |
|  | *Predator* | Field-level application of pollen promote whitefly control by enhancing populations of predatory mites |
|  | *Inter. predators& parasitoids* | The predators *Macrolophus pygmaeus* and *Nesidiocoris tenuis* and the parasitoid *Eretmocerus mundus* are highly efficacious in controlling *B. tabaci* populations |
|  | *Biopesticides* | Essential oils from the leaves of prickly ash (*Zanthoxylum rhoifolium*) contain nanoparticles, which can be further prepared through nanoprecipitation and encapsulation |
|  | *Biopesticides* | Essential oil derived from garden thyme *Thymus vulgaris* or patchouli *Pogostemon cablin* exerted contact toxicity or repellency on *B. tabaci*, and can thus be used for the production of bio-pesticides |

a PRACT=is widely used in the country; RESEAR= at research state only; READY=IPM alternative available for immediate implementation; b frequently listed roadblocks, preventing technology diffusion: ENVI= It would not be effective under the environmental conditions of the country; ECON= deemed too expensive - so it is not widely adopted

Table 8b Pest management alternatives identified through the literature review for silver-leaf whitefly (*Bemisia tabaci*) in cotton

| Categories | Sub-categories | IPM alternatives identified |
| --- | --- | --- |
|  |  |  |
| Biological control | *Biopesticides* | The use of NeemAzal® by soil drenching resulted in stable effects under both laboratory and greenhouse conditions, and helps achieve high efficiency and persistence as compared to above-ground applications |
|  | *Biopesticides* | Botanical and mineral oils are highly effective against whitefly nymphs, and somewhat effective against adults within 24 h of spraying – yet the latter show low foliar persistence |
|  | *Biopesticides* | Oils from five different vegetable seeds –i.e., peanut, cottonseed, castor, soybean and sunflower – had insecticidal effects on adult and immature stages of B. tabaci, and equally affected settling and oviposition deterrence, thus showing ample promise as direct and residual larvicides |
|  | *Biopesticides* | Essential oils from *Pelargonium graveolens* reduced the number of *B. tabaci* adults |
|  | *Biopesticides* | Extracts of medicinal plants such as *Rutac halepensis*, *Peganum harmala* and *Alkanna strigosa* reduced numbers of *B. tabaci* immatures to similar degree as insecticidal sprays, yet did not negatively affect the parasitoid *Eretmocerus mundus*; plant extracts from *Urtica pilulifera* and *T. capita* were repellent |
|  | *CBC* | In-field abundance of parasitoids can be enhanced establishing *Lantana camara, Bauhinia pupurea, Albizzia lebbek and Morus alba* in or near cotton |
|  | *CBC* | Several weeds – i.e., common ragweed, velvetleaf – are colonized earlier by *B. tabaci* than cotton, and common ragweed supports 12-22 fold higher whitefly population densities than cotton; sunflower supported 1.5-2 fold more whitefly than cotton did. Hence, certain weeds can increase whitefly population density on cotton, while others -including sunflower- can act as a trap crop |
| Cultural or mechanical control | *Cultural practices* | The establishment of host-free periods and various forms of screened (physical) exclusion can assist in whitefly management |
|  | *Nutrition managem.* | N fertilization rates and peak infestation pressure of adult and immature whiteflies are directly related; balanced N application is therefore recommended |
|  | *Water managem.* | In furrow-irrigated fields, significantly higher numbers of whitefly immatures were recorded than in drip-irrigated plots; whitefly population numbers were highest in a 33% (1/3) water supply regime and *B. tabaci* performance was superior on water-stressed plants |
|  | *Trap crop* | In field trials, *B. tabaci* egg and nymph densities were >10-fold higher on cantaloupe than cotton; establishment of a cantaloupe trap crop lowered whitefly densities in the cotton field but those were not below established economic thresholds. Yet, cantaloupe trap cropping can be part of an IPM strategy |
|  | *Trap crop* | In cotton fields with (insecticide-treated) strips of velvetleaf (*Abutilon theophrasti*), densities of B. tabaci were 21–51 % lower than in those without |
| DSS |  | A multiple-threshold-based management and sampling plan was developed and validated; control options based on application of insect growth regulators (IGRs) are recommended for high-density populations whereas conventional (non-IGR) products are recommended for the control of low to moderate population densities. Binomial models with thresholds of T=1, 2 and 3 adults/leaf were tested using the field abundance data. A binomial plan is recommended as sampling protocol for Australian cotton fields. A decision support guide with management zone boundaries expressed as binomial counts and control options appropriate for various *B. tabaci* density situations |

a PRACT=is widely used in the country; RESEAR= at research state only; READY=IPM alternative available for immediate implementation; b frequently listed roadblocks, preventing technology diffusion: ENVI= It would not be effective under the environmental conditions of the country; ECON= deemed too expensive - so it is not widely adopted

Table 8c Pest management alternatives identified through the literature review for silver-leaf whitefly (*Bemisia tabaci*) in cotton

| Categories | Sub-categories | IPM alternatives identified |
| --- | --- | --- |
|  |  |  |
| Other | *IPM* | An IPM model that organizes all *B. tabaci* control tactics into a multi-level, multi-component pyramid built around three core components: “sampling”, “effective chemical use”, and “avoidance”; the concept “bioresidual” reflects extended periods of *B. tabaci* suppression possible through proper use of IGRs |
|  | *IPM* | Following (large-scale) establishment of GM cotton, (sap-feeding) pest species have gained in importance and pests, such as *B. tabaci*, are progressively displacing caterpillars as key pests of *Bt* cotton. Yet, systems-level interventions such as the maintenance of permanent ground cover, intercropping and trap cropping have proven effective in favoring arthropod natural enemies and thus slowing whitefly population build-up |
| Innov. pestic. and appl. reg. | *Active ingredient (AI)* | Applications of milbemectin helped control *B. tabaci* populations, with their efficacy further enhanced through the addition of mineral oil, yet they do not negatively impact whitefly natural enemies of *B. tabaci* |
|  | *Appl. techn.* | Upon use as foliar sprays, different pesticides reduced significantly more sucking pests, including *B. tabaci*, than when used as drench |
|  | *Anti-resist. strategy* | Insecticide resistance management strategies based on rational use of products with new modes of action, coupled with enhanced implementation of cultural management and biological control constitutes the best approach for combating insecticide resistance in *B. tabaci* whitefly |
|  | *Synergist* | Piperonyl butoxide (PBO) effected good control over *B. tabaci* nymphs, and neither PBO, buprofezin nor the oil negatively impacted natural enemies |
|  | *Surfactants* | Isiloxane-based surfactants (Break-thru® and Silwet® L-77) exhibited the highest toxicity to 1st–2nd (early) and 3rd–4th (late) instar nymphs as well as the greatest wetting performance |
| Host plant resistance | *Resistance* | The expression of insecticidal proteins and/or toxins or double-stranded (ds)RNA homologous to *B. tabaci* genes offers protection against *B. tabaci* and the viruses that it transmits |
|  | *Resistance* | Different traits were identified linked to host plant resistance to *B. tabaci* whitefly, as such: okra leaf traits reduced adult preference, glabrous leaf traits reduced oviposition preference, and bio-chemical traits in *Gossypium arboreum* lowered immature development and/or survival; traits could be further incorporated in breeding programs for whitefly-resistant upland cotton cultivars |
|  | *Resistance* | Upon evaluation of 12 cotton genotypes, a strong tolerance mechanism is operating in NHH 44, and genotypes LD 694 and PA 183 are rated as moderately tolerant |
|  | *Resistance* | Upon evaluation of different genotypes, LD694 is rated as resistant in three consecutive generations of whitefly; LK861, Supriya, RS2013, CNH911 and PA183 as moderately resistant, and IS-376/4/1/20/72, NHH44, TxMaroon2-78, Bt 6304 and RS2098 as moderately susceptible |

a PRACT=is widely used in the country; RESEAR= at research state only; READY=IPM alternative available for immediate implementation; b frequently listed roadblocks, preventing technology diffusion: ENVI= It would not be effective under the environmental conditions of the country; ECON= deemed too expensive - so it is not widely adopted

**Fig. 4**. Geographical distribution of data on alternative tools identified through the literature review (Europe). Only field data are presented (visit the website <https://drive.google.com/open?id=1Ce7jYMiMScXZCU8ynX6KBGaSYVFsi70o&usp=sharing>)

**Fig. 5.** Geographical distribution of data on alternative tools identified through the literature review (outside Europe). Only field data are presented (visit the website: <https://drive.google.com/open?id=1ucgASk2MTTfPPq5W6lKYG95izmXZqTjV&usp=sharing> )

List of references included as data in the literature review

Ahmad, Mushtaq, and M. Iqbal Arif. 2008. "Susceptibility of Pakistani populations of cotton aphid Aphis gossypii (Homoptera: Aphididae) to endosulfan, organophosphorus and carbamate insecticides." *Crop Protection* 27 (3):523-531. doi: https://doi.org/10.1016/j.cropro.2007.08.006.

Ai, Tian-Cheng, Zhang-Yong Liu, Chuan-Ren Li, Pan Luo, Jian-Qiang Zhu, Wei-Bing Jin, and Qing-Nian Cai. 2011. "Impact of fertilization on cotton aphid population in Bt-cotton production system." *Ecological Complexity* 8 (1):9-14. doi: https://doi.org/10.1016/j.ecocom.2010.08.002.

Al-mazra’awi, Mohammad S., and Mazen Ateyyat. 2009. "Insecticidal and repellent activities of medicinal plant extracts against the sweet potato whitefly, Bemisia tabaci (Hom.: Aleyrodidae) and its parasitoid Eretmocerus mundus (Hym.: Aphelinidae)." *Journal of Pest Science* 82 (2):149-154. doi: 10.1007/s10340-008-0233-x.

Alignier, Audrey, Lucie Raymond, Marc Deconchat, Philippe Menozzi, Claude Monteil, Jean-Pierre Sarthou, Aude Vialatte, and Annie Ouin. 2014. "The effect of semi-natural habitats on aphids and their natural enemies across spatial and temporal scales." *Biological Control* vol. 77:pp. 76-82. doi: 10.1016/j.biocontrol.2014.06.006.

Ansari, M. A., M. Evans, and T. M. Butt. 2009. "Identification of pathogenic strains of entomopathogenic nematodes and fungi for wireworm control." *Crop Protection* 28 (3):269-272. doi: https://doi.org/10.1016/j.cropro.2008.11.003.

Antony, B., M. S. Palaniswami, A. A. Kirk, and T. J. Henneberry. 2004. "Development of Encarsia bimaculata (Heraty and Polaszek) (Hymenoptera: Aphelinidae) in Bemisia tabaci (Gennadius) (Homoptera: Aleyrodidae) nymphs." *Biological Control* 30 (3):546-555. doi: https://doi.org/10.1016/j.biocontrol.2004.01.018.

Aqueel, M. A., and S. R. Leather. 2011. "Effect of nitrogen fertilizer on the growth and survival of Rhopalosiphum padi (L.) and Sitobion avenae (F.) (Homoptera: Aphididae) on different wheat cultivars." *Crop Protection* 30 (2):216-221. doi: https://doi.org/10.1016/j.cropro.2010.09.013.

Baldin, Edson L. L., Gabriela P. Aguiar, Thiago L. M. Fanela, Muriel C. E. Soares, Milton Groppo, and Antônio E. M. Crotti. 2015. "Bioactivity of Pelargonium graveolens essential oil and related monoterpenoids against sweet potato whitefly, Bemisia tabaci biotype B." *Journal of Pest Science* 88 (1):191-199. doi: 10.1007/s10340-014-0580-8.

Barsics, Fanny, Benjamin M. Delory, Pierre Delaplace, Frédéric Francis, Marie-Laure Fauconnier, Éric Haubruge, and François J. Verheggen. 2017. "Foraging wireworms are attracted to root-produced volatile aldehydes." *Journal of Pest Science* 90 (1):69-76. doi: 10.1007/s10340-016-0734-y.

Basanth, Y. S., V. T. Sannaveerappanavar, and D. K. Sidde Gowda. 2013. "Susceptibility of Different Populations of Nilaparvata lugens from Major Rice Growing Areas of Karnataka, India to Different Groups of Insecticides." *Rice Science* 20 (5):371-378. doi: https://doi.org/10.1016/S1672-6308(13)60147-X.

Bellamy, David E., Mark K. Asplen, and David N. Byrne. 2004. "Impact of Eretmocerus eremicus (Hymenoptera: Aphelinidae) on open-field Bemisia tabaci (Hemiptera: Aleyrodidae) populations." *Biological Control* 29 (2):227-234. doi: https://doi.org/10.1016/S1049-9644(03)00150-6.

Benefer, Carly M., Mairi E. Knight, Jonathan S. Ellis, Helen Hicks, and Rod P. Blackshaw. 2012. "Understanding the relationship between adult and larval Agriotes distributions: The effect of sampling method, species identification and abiotic variables." *Applied Soil Ecology* 53:39-48. doi: https://doi.org/10.1016/j.apsoil.2011.11.004.

Bestete, Luziani R., Jorge B. Torres, Rebecca B. B. Silva, Christian S. A. Silva-Torres, and Cristina S. Bastos. 2017. "Development of cotton pests exhibiting different feeding strategy on water-stressed and kaolin-treated cotton plants." *Journal of Pest Science* 90 (1):139-150. doi: 10.1007/s10340-016-0773-4.

Bi, J. L., N. C. Toscano, and M. A. Madore. 2003. "Effect of Urea Fertilizer Application on Soluble Protein and Free Amino Acid Content of Cotton Petioles in Relation to Silverleaf Whitefly (Bemisia argentifolii) Populations." *Journal of Chemical Ecology* 29 (3):747-761. doi: 10.1023/a:1022880905834.

Brandl, M. A., M. Schumann, B. W. French, and S. Vidal. 2016. "Screening of Botanical Extracts for Repellence against Western Corn Rootworm Larvae." *Journal of Insect Behavior* 29 (4):395-414. doi: 10.1007/s10905-016-9571-3.

Brandl, M. A., M. Schumann, M. Przyklenk, A. Patel, and S. Vidal. 2017. "Wireworm damage reduction in potatoes with an attract-and-kill strategy using Metarhizium brunneum." *Journal of Pest Science* 90 (2):479-493. doi: 10.1007/s10340-016-0824-x.

Cabanillas, H. Enrique, and Walker A. Jones. 2009. "Pathogenicity of Isaria sp. (Hypocreales: Clavicipitaceae) against the sweet potato whitefly B biotype, Bemisia tabaci (Hemiptera: Aleyrodidae)." *Crop Protection* 28 (4):333-337. doi: https://doi.org/10.1016/j.cropro.2008.11.015.

Cao Quoc, Nam, Nico Vromant, Be Tran Thanh, and Frans Ollevier. 2012. "Investigation of the predation potential of different fish species on brown planthopper (Nilaparvata lugens (Stål)) in experimental rice-fish aquariums and tanks." *Crop Protection* 38:95-102. doi: https://doi.org/10.1016/j.cropro.2012.03.003.

Capra, Emanuele, Cinzia Colombi, Pamela De Poli, Fabio Francesco Nocito, Maurizio Cocucci, Alberto Vecchietti, Adriano Marocco, Maria Rosaria Stile, and Laura Rossini. 2015. "Protein profiling and tps23 induction in different maize lines in response to methyl jasmonate treatment and Diabrotica virgifera infestation." *Journal of Plant Physiology* 175:68-77. doi: https://doi.org/10.1016/j.jplph.2014.10.018.

Castle, S. J. 2006. "Concentration and management of Bemisia tabaci in cantaloupe as a trap crop for cotton." *Crop Protection* 25 (6):574-584. doi: https://doi.org/10.1016/j.cropro.2005.08.013.

Choate, Beth A., and Jonathan G. Lundgren. 2015. "Invertebrate communities in spring wheat and the identification of cereal aphid predators through molecular gut content analysis." *Crop Protection* 77:110-118. doi: https://doi.org/10.1016/j.cropro.2015.07.021.

Christofoli, Marcela, Eliangela Cristina Candida Costa, Keylla U. Bicalho, Vanessa de Cássia Domingues, Márcio Fernandes Peixoto, Cassia Cristina Fernandes Alves, Wagner L. Araújo, and Cristiane de Melo Cazal. 2015. "Insecticidal effect of nanoencapsulated essential oils from Zanthoxylum rhoifolium (Rutaceae) in Bemisia tabaci populations." *Industrial Crops and Products* 70:301-308. doi: https://doi.org/10.1016/j.indcrop.2015.03.025.

Chrzanowski, Grzegorz, Bogumił Leszczyński, Paweł Czerniewicz, Hubert Sytykiewicz, Henryk Matok, Robert Krzyżanowski, and Cezary Sempruch. 2012. "Effect of phenolic acids from black currant, sour cherry and walnut on grain aphid (Sitobion avenae F.) development." *Crop Protection* 35:71-77. doi: https://doi.org/10.1016/j.cropro.2012.01.005.

Cossé, Allard A., and Thomas C. Baker. 1999. "Electrophysiologically and Behaviorally Active Volatiles of Buffalo Gourd Root Powder for Corn Rootworm Beetles." *Journal of Chemical Ecology* 25 (1):51-66. doi: 10.1023/a:1020885032397.

Crafts-Brandner, S. J. 2002. "Plant nitrogen status rapidly alters amino acid metabolism and excretion in Bemisia tabaci." *Journal of Insect Physiology* 48 (1):33-41. doi: https://doi.org/10.1016/S0022-1910(01)00140-8.

Dang, Quang Le, Yong Ho Choi, Gyung Ja Choi, Kyoung Soo Jang, Myung Soo Park, No-Joong Park, Chi Hwan Lim, Hyungrok Kim, Luu Hoang Ngoc, and Jin-Cheol Kim. 2010. "Pesticidal activity of ingenane diterpenes isolated from Euphorbia kansui against Nilaparvata lugens and Tetranychus urticae." *Journal of Asia-Pacific Entomology* 13 (1):51-54. doi: https://doi.org/10.1016/j.aspen.2009.09.002.

Dang, Quang Le, Gee Young Lee, Yong Ho Choi, Gyung Ja Choi, Kyoung Soo Jang, Myung Soo Park, Ho Seob Soh, Young Hee Han, Chi Hwan Lim, and Jin-Cheol Kim. 2010. "Insecticidal activities of crude extracts and phospholipids from Chenopodium ficifolium against melon and cotton aphid, Aphis gossypii." *Crop Protection* 29 (10):1124-1129. doi: https://doi.org/10.1016/j.cropro.2010.06.009.

de Almeida Marques, Míriam, Eliane Dias Quintela, Gabriel Moura Mascarin, Paulo Marçal Fernandes, and Steven Paul Arthurs. 2014. "Management of Bemisia tabaci biotype B with botanical and mineral oils." *Crop Protection* 66:127-132. doi: https://doi.org/10.1016/j.cropro.2014.09.006.

Dedryver, Charles-Antoine, Anne Le Ralec, and Frédéric Fabre. 2010. "The conflicting relationships between aphids and men: A review of aphid damage and control strategies." *Comptes Rendus Biologies* 333 (6):539-553. doi: https://doi.org/10.1016/j.crvi.2010.03.009.

Devine, Gregor J., Isaac Ishaaya, A. Rami Horowitz, and Ian Denholm. 1998. "Effects of piperonyl butoxide on Bemisia tabaci Genn. (Homoptera: Aleyrodidae): mortality, development, parasitism and predation in Israeli cotton fields." *Crop Protection* 17 (9):717-726. doi: https://doi.org/10.1016/S0261-2194(98)00080-5.

Dogimont, Catherine, Abdelhafid Bendahmane, Véronique Chovelon, and Nathalie Boissot. 2010. "Host plant resistance to aphids in cultivated crops: Genetic and molecular bases, and interactions with aphid populations." *Comptes Rendus Biologies* 333 (6):566-573. doi: https://doi.org/10.1016/j.crvi.2010.04.003.

Down, Rachel E., Andrew G. S. Cuthbertson, James J. Mathers, and Keith F. A. Walters. 2009. "Dissemination of the entomopathogenic fungi, Lecanicillium longisporum and L. muscarium, by the predatory bug, Orius laevigatus, to provide concurrent control of Myzus persicae, Frankliniella occidentalis and Bemisia tabaci." *Biological Control* 50 (2):172-178. doi: https://doi.org/10.1016/j.biocontrol.2009.03.010.

Ellsworth, Peter C., and Jose Luis Martinez-Carrillo. 2001. "IPM for Bemisia tabaci: a case study from North America." *Crop Protection* 20 (9):853-869. doi: https://doi.org/10.1016/S0261-2194(01)00116-8.

Erb, Matthias, Gaetan Glauser, and Christelle A. M. Robert. 2012. "Induced Immunity Against Belowground Insect Herbivores- Activation of Defenses in the Absence of a Jasmonate Burst." *Journal of Chemical Ecology* 38 (6):629-640. doi: 10.1007/s10886-012-0107-9.

Fabre, Frédéric, Charles-Antoine Dedryver, Manuel Plantegenest, Maurice Hullé, and Etienne Rivot. 2010. "Hierarchical Bayesian Modelling of plant colonisation by winged aphids: Inferring dispersal processes by linking aerial and field count data." *Ecological Modelling* 221 (15):1770-1778. doi: https://doi.org/10.1016/j.ecolmodel.2010.04.006.

Faria, Marcos, and Stephen P. Wraight. 2001. "Biological control of Bemisia tabaci with fungi." *Crop Protection* 20 (9):767-778. doi: https://doi.org/10.1016/S0261-2194(01)00110-7.

Fenigstein, Annie, Miriam Eliyahu, S. Gan-Mor, and D. Veierov. 2001. "Effects of five vegetable oils on the sweetpotato whiteflyBemisia tabaci." *Phytoparasitica* 29 (3):197-206. doi: 10.1007/bf02983451.

Fishilevich, Elane, Ana M. Vélez, Chitvan Khajuria, Meghan L. F. Frey, Ronda L. Hamm, Haichuan Wang, Greg A. Schulenberg, Andrew J. Bowling, Heather E. Pence, Premchand Gandra, Kanika Arora, Nicholas P. Storer, Kenneth E. Narva, and Blair D. Siegfried. 2016. "Use of chromatin remodeling ATPases as RNAi targets for parental control of western corn rootworm (Diabrotica virgifera virgifera) and Neotropical brown stink bug (Euschistus heros)." *Insect Biochemistry and Molecular Biology* 71:58-71. doi: https://doi.org/10.1016/j.ibmb.2016.02.004.

Furlan, L., Vasileios P. Vasileiadis, Francesca Chiarini, Hilfred Huiting, Robert Leskovšek, Jaka Razinger, Imre J. Holb, Erica Sartori, Gregor Urek, Arnd Verschwele, Isadora Benvegnù, and Maurizio Sattin. 2017. "Risk assessment of soil-pest damage to grain maize in Europe within the framework of Integrated Pest Management." *Crop Protection* 97:52-59. doi: https://doi.org/10.1016/j.cropro.2016.11.029.

Furlan, Lorenzo. 2014. "IPM thresholds for Agriotes wireworm species in maize in Southern Europe." *Journal of Pest Science* 87 (4):609-617. doi: 10.1007/s10340-014-0583-5.

Furlan, Lorenzo, Christian Bonetto, Andrea Finotto, Luca Lazzeri, Lorena Malaguti, Giampiero Patalano, and William Parker. 2010. "The efficacy of biofumigant meals and plants to control wireworm populations." *Industrial Crops and Products* 31 (2):245-254. doi: https://doi.org/10.1016/j.indcrop.2009.10.012.

Gencsoylu, I., A. R. Horowitz, F. Sezgin, and C. Oncüer. 2003. "Effect of drip and furrow irrigation methods onBemisia tabaci populations in cotton fields." *Phytoparasitica* 31 (2):139-143. doi: 10.1007/bf02980783.

Gerling, Dan, and Steven E. Naranjo. 1998. "The Effect of Insecticide Treatments in Cotton Fields on the Levels of Parasitism ofBemisia tabaci(Gennadius)sl." *Biological Control* 12 (1):33-41. doi: https://doi.org/10.1006/bcon.1998.0613.

Gfeller, Aurélie, Morgan Laloux, Fanny Barsics, Djamel Edine Kati, Eric Haubruge, Patrick du Jardin, François J. Verheggen, Georges Lognay, Jean-Paul Wathelet, and Marie-Laure Fauconnier. 2013. "Characterization of Volatile Organic Compounds Emitted by Barley (Hordeum vulgare L.) Roots and Their Attractiveness to Wireworms." *Journal of Chemical Ecology* 39 (8):1129-1139. doi: 10.1007/s10886-013-0302-3.

Giarola, L. T. P., S. G. F. Martins, and M. C. P. Toledo Costa. 2006. "Computer simulation of Aphis gossypii insects using Penna aging model." *Physica A: Statistical Mechanics and its Applications* 368 (1):147-154. doi: https://doi.org/10.1016/j.physa.2005.11.057.

Gindin, G., N. U. Geschtovt, B. Raccah, and I. Barash. 2000. "Pathogenicity ofVerticillium lecanii to different developmental stages of the silverleaf whitefly,Bemisia argentifolii." *Phytoparasitica* 28 (3):229. doi: 10.1007/bf02981801.

Gonzalez-Andujar, J. L., J. L. Garcia-de Ceca, and A. Fereres. 1993. "Cereal aphids expert system (CAES): Identification and decision making." *Computers and Electronics in Agriculture* 8 (4):293-300. doi: https://doi.org/10.1016/0168-1699(93)90017-U.

Gosselke, U., H. Triltsch, D. Roßberg, and B. Freier. 2001. "GETLAUS01—the latest version of a model for simulating aphid population dynamics in dependence on antagonists in wheat." *Ecological Modelling* 145 (2):143-157. doi: https://doi.org/10.1016/S0304-3800(01)00386-6.

Griffiths, D C. 1974. "Susceptibility of plants to attack by wireworms (Agriotes spp.)." *Annals of Applied Biology* 78 (1):7-13. doi: doi:10.1111/j.1744-7348.1974.tb01480.x.

Gurulingappa, Pampapathy, Gregory A. Sword, Gregory Murdoch, and Peter A. McGee. 2010. "Colonization of crop plants by fungal entomopathogens and their effects on two insect pests when in planta." *Biological Control* 55 (1):34-41. doi: https://doi.org/10.1016/j.biocontrol.2010.06.011.

Hadi, Buyung A. R., Charle Patrick F. Garcia, and K. L. Heong. 2015. "Susceptibility of Nilaparvata lugens (Hemipteran: Delphacidae) populations in the Philippines to insecticides." *Crop Protection* 76:100-102. doi: https://doi.org/10.1016/j.cropro.2015.07.002.

Hamada, A. M., and L. M. Jonsson. 2013. "Thiamine treatments alleviate aphid infestations in barley and pea." *Phytochemistry* 94:135-41. doi: 10.1016/j.phytochem.2013.05.012.

Hammack, Leslie. 1996. "Corn volatiles as attractants for northern and western corn rootworm beetles (Coleoptera: Chrysomelidae:Diabrotica spp.)." *Journal of Chemical Ecology* 22 (7):1237-1253. doi: 10.1007/bf02266963.

Hammack, Leslie. 2001. "Single and Blended Maize Volatiles as Attractants for Diabroticite Corn Rootworm Beetles." *Journal of Chemical Ecology* 27 (7):1373-1390. doi: 10.1023/a:1010365225957.

Hammack, Leslie, and Richard J. Petroski. 2004. "Field Capture of Northern and Western Corn Rootworm Beetles Relative to Attractant Structure and Volatility." *Journal of Chemical Ecology* 30 (9):1809-1825. doi: 10.1023/B:JOEC.0000042403.88930.a7.

He, Wenqiang, Meng Yang, Zhihua Li, Junli Qiu, Fang Liu, Xiaosheng Qu, Yongfu Qiu, and Rongbai Li. 2015. "High levels of silicon provided as a nutrient in hydroponic culture enhances rice plant resistance to brown planthopper." *Crop Protection* 67:20-25. doi: https://doi.org/10.1016/j.cropro.2014.09.013.

Hermann, A., N. Brunner, P. Hann, T. Wrbka, and B. Kromp. 2013. "Correlations between wireworm damages in potato fields and landscape structure at different scales." *Journal of Pest Science* 86 (1):41-51. doi: 10.1007/s10340-012-0444-z.

Hibbard, B. E., and L. B. Bjostad. 1988. "Behavioral responses of western corn rootworm larvae to volatile semiochemicals from corn seedlings." *Journal of Chemical Ecology* 14 (6):1523-1539. doi: 10.1007/bf01012424.

Hibbard, Bruce E., Elisa J. Bernklau, and Louis B. Bjostad. 1994. "Long-chain free fatty acids: Semiochemicals for host location by western corn rootworm larvae." *Journal of Chemical Ecology* 20 (12):3335. doi: 10.1007/bf02033730.

Hibbard, Bruce E., and Louis B. Bjostad. 1990. "Isolation of corn semiochemicals attractive and repellent to western corn rootworm larvae." *Journal of Chemical Ecology* 16 (12):3425-3439. doi: 10.1007/bf00982108.

Hicks, Helen, and Rod P. Blackshaw. 2008. "Differential responses of three Agriotes click beetle species to pheromone traps." *Agricultural and Forest Entomology* 10 (4):443-448. doi: doi:10.1111/j.1461-9563.2008.00397.x.

Hoelmer, K. A. 2007. "Field cage evaluation of introduced Eretmocerus species (Hymenoptera: Aphelinidae) against Bemisia tabaci strain B (Homoptera: Aleyrodidae) on cantaloupe." *Biological Control* 43 (2):156-162. doi: https://doi.org/10.1016/j.biocontrol.2007.07.010.

Holland, John, H. Oaten, S. Southway, and Stephen Moreby. 2008. *The effectiveness of field margin enhancement for cereal aphid control by different natural enemy guilds*. Vol. 47.

Hong-xing, Xu, Yang Ya-jun, Lu Yan-hui, Zheng Xu-song, Tian Jun-ce, Lai Feng-xiang, Fu Qiang, and Lu Zhong-xian. 2017. "Sustainable Management of Rice Insect Pests by Non-Chemical-Insecticide Technologies in China." *Rice Science* 24 (2):61-72. doi: https://doi.org/10.1016/j.rsci.2017.01.001.

Horgan, Finbarr G., Angelee Fame Ramal, Jagadish S. Bentur, Ram Kumar, K. Vasanta Bhanu, Preetinder Singh Sarao, Eko Hari Iswanto, Ho Van Chien, Moe Hnin Phyu, Carmencita C. Bernal, Maria Liberty P. Almazan, Mohammad Zahangeer Alam, Zhongxian Lu, and Shou-Horng Huang. 2015. "Virulence of brown planthopper (Nilaparvata lugens) populations from South and South East Asia against resistant rice varieties." *Crop Protection* 78:222-231. doi: https://doi.org/10.1016/j.cropro.2015.09.014.

Hu, Xiang-shun, Hui-yan Zhao, Zu-qing Hu, Dong-hong Li, and Yu-hong Zhang. 2008. "EPG Comparison of Sitobion avenae (Fab.) Feeding Behavior on Three Wheat Varieties." *Agricultural Sciences in China* 7 (2):180-186. doi: https://doi.org/10.1016/S1671-2927(08)60037-1.

Jaffuel, Geoffrey, Ivan Hiltpold, and Ted C. J. Turlings. 2015. "Highly Potent Extracts from Pea (Pisum sativum) and Maize (Zea mays) Roots Can Be Used to Induce Quiescence in Entomopathogenic Nematodes." *Journal of Chemical Ecology* 41 (9):793-800. doi: 10.1007/s10886-015-0623-5.

Jedlička, Pavel, and Jan Frouz. 2007. "Population dynamics of wireworms (Coleoptera, Elateridae) in arable land after abandonment." *Biologia* 62 (1):103-111. doi: 10.2478/s11756-007-0017-4.

Jeon, Ju-Hyun, Yeon-Kook Kim, Sang-Guei Lee, Geon-Hwi Lee, and Hoi-Seon Lee. 2011. "Insecticidal activities of a Diospyros kaki root-isolated constituent and its derivatives against Nilaparvata lugens and Laodelphax striatellus." *Journal of Asia-Pacific Entomology* 14 (4):449-453. doi: https://doi.org/10.1016/j.aspen.2011.07.005.

Ji, Zhi-juan, Shu-dong Yang, Yu-xiang Zeng, Yan Liang, Chang-deng Yang, and Qian Qian. 2016. "Pyramiding blast, bacterial blight and brown planthopper resistance genes in rice restorer lines." *Journal of Integrative Agriculture* 15 (7):1432-1440. doi: https://doi.org/10.1016/S2095-3119(15)61165-0.

Johnson, Scott N., Carly M. Benefer, Adam Frew, Bryan S. Griffiths, Susan E. Hartley, Alison J. Karley, Sergio Rasmann, Mario Schumann, Illja Sonnemann, and Christelle A. M. Robert. 2016. "New frontiers in belowground ecology for plant protection from root-feeding insects." *Applied Soil Ecology* 108:96-107. doi: https://doi.org/10.1016/j.apsoil.2016.07.017.

Jung, Jeanette, Paolo Racca, Juliane Schmitt, and Benno Kleinhenz. 2014. "SIMAGRIO‐W: Development of a prediction model for wireworms in relation to soil moisture, temperature and type." *Journal of Applied Entomology* 138 (3):183-194. doi: doi:10.1111/jen.12021.

Kabaluk, Todd. 2014. "Targeting the click beetle Agriotes obscurus with entomopathogens as a concept for wireworm biocontrol." *BioControl* 59 (5):607-616. doi: 10.1007/s10526-014-9603-x.

Khatiwada, Janak Raj, Subarna Ghimire, Shanta Paudel Khatiwada, Bikash Paudel, Richard Bischof, Jianping Jiang, and Torbjørn Haugaasen. 2016. "Frogs as potential biological control agents in the rice fields of Chitwan, Nepal." *Agriculture, Ecosystems & Environment* 230:307-314. doi: https://doi.org/10.1016/j.agee.2016.06.025.

Kim, Jeong Jun, Mark S. Goettel, and David R. Gillespie. 2008. "Evaluation of Lecanicillium longisporum, Vertalec® for simultaneous suppression of cotton aphid, Aphis gossypii, and cucumber powdery mildew, Sphaerotheca fuliginea, on potted cucumbers." *Biological Control* 45 (3):404-409. doi: https://doi.org/10.1016/j.biocontrol.2008.02.003.

Kim, Jeong Jun, and Kyu Chin Kim. 2008. "Selection of a highly virulent isolate of Lecanicillium attenuatum against cotton aphid." *Journal of Asia-Pacific Entomology* 11 (1):1-4. doi: https://doi.org/10.1016/j.aspen.2008.02.001.

Kleespies, Regina G., Claudia Ritter, Gisbert Zimmermann, Frank Burghause, Simon Feiertag, and Andreas Leclerque. 2013. "A survey of microbial antagonists of Agriotes wireworms from Germany and Italy." *Journal of Pest Science* 86 (1):99-106. doi: 10.1007/s10340-012-0447-9.

Koo, Hyun-Na, Jeong-Jin An, Sang-Eun Park, Ju-Il Kim, and Gil-Hah Kim. 2014. "Regional susceptibilities to 12 insecticides of melon and cotton aphid, Aphis gossypii (Hemiptera: Aphididae) and a point mutation associated with imidacloprid resistance." *Crop Protection* 55:91-97. doi: https://doi.org/10.1016/j.cropro.2013.09.010.

Korinsak, Siriporn, Meechai Siangliw, Jate Kotcharerk, Jirapong Jairin, Jonaliza L. Siangliw, Boonrat Jongdee, Grienggrai Pantuwan, Nitat Sidthiwong, and Theerayut Toojinda. 2016. "Improvement of the submergence tolerance and the brown planthopper resistance of the Thai jasmine rice cultivar KDML105 by pyramiding Sub1 and Qbph12." *Field Crops Research* 188:105-112. doi: https://doi.org/10.1016/j.fcr.2015.10.025.

Kumar, Prabhat. 2008. "Studies on loss of bio-efficacy of two indirect neem application over time (seed and soil) against Bemisia tabaci (Homoptera: Aleyrodidae) under semi-field conditions." *Journal of Asia-Pacific Entomology* 11 (4):185-190. doi: https://doi.org/10.1016/j.aspen.2008.08.003.

Kumar, Rishi, S. Kranthi, M. Nitharwal, S. L. Jat, and D. Monga. 2012. "Influence of pesticides and application methods on pest and predatory arthropods associated with cotton." *Phytoparasitica* 40 (5):417-424. doi: 10.1007/s12600-012-0241-5.

Kuusk, A. K., A. Cassel-Lundhagen, A. Kvarnheden, and B. Ekbom. 2008. "Tracking aphid predation by lycosid spiders in spring-sown cereals using PCR-based gut-content analysis." *Basic and Applied Ecology* 9 (6):718-725. doi: https://doi.org/10.1016/j.baae.2007.08.012.

Kwon, Soon Hwa, and Doog-Soon Kim. 2017. "Effects of temperature and photoperiod on the production of sexual morphs of Aphis gossypii (Hemiptera: Aphididae) in Jeju, Korea." *Journal of Asia-Pacific Entomology* 20 (1):53-56. doi: https://doi.org/10.1016/j.aspen.2016.11.006.

Langer, Alain, and Thierry Hance. 2004. "Enhancing parasitism of wheat aphids through apparent competition: a tool for biological control." *Agriculture, Ecosystems & Environment* 102 (2):205-212. doi: https://doi.org/10.1016/j.agee.2003.07.005.

Lazreg, Fatiha, Zhen Huang, Shaukat Ali, and Shunxiang Ren. 2009. "Effect of Lecanicillium muscarium on Eretmocerus sp. nr. furuhashii (Hymenoptera: Aphelinidae), a parasitoid of Bemisia tabaci (Hemiptera: Aleyrodidae)." *Journal of Pest Science* 82 (1):27-32. doi: 10.1007/s10340-008-0215-z.

Leclerque, Andreas, Polina V. Mitkovets, Ana-Cristina Fatu, Christina Schuster, and Regina G. Kleespies. 2013. "Ribosomal RNA phylogeny of bacterial and fungal pathogens of Agriotes wireworms." *Journal of Pest Science* 86 (1):107-113. doi: 10.1007/s10340-012-0450-1.

Lee, Hoi-Scon, Wook-Kyun Shin, Cheol Song, Kwang-Yun Cho, and Young-Joon Ahn. 2001. "Insecticidal Activities of ar-Turmerone Identified in Curcuma longa Rhizome against Nilaparvata lugens (Homoptera: Delphacidae) and Plutella xylostella (Lepidoptera: Yponomeutidae)." *Journal of Asia-Pacific Entomology* 4 (2):181-185. doi: https://doi.org/10.1016/S1226-8615(08)60121-1.

Lin, Kejian, Yanhui Lu, Peng Wan, Yizhong Yang, Kris A. G. Wyckhuys, and Kongming Wu. 2015. "Simultaneous reduction in incidence of Bemisia tabaci (Hemiptera: Aleyrodidae) and Sylepta derogata (Lepidoptera: Pyralidae) using velvetleaf, Abutilon theophrasti as a trap crop." *Journal of Pest Science* 88 (1):49-56. doi: 10.1007/s10340-014-0584-4.

Lin, S., M. S. You, G. Yang, and L. L. Chen. 2011. "Can polycultural manipulation effectively control rice planthoppers in rice-based ecosystems?" *Crop Protection* 30 (3):279-284. doi: https://doi.org/10.1016/j.cropro.2010.11.013.

Liu, Jing-Lan, Hong-Mei Zhang, Xiao Chen, Xia Yang, and Jin-Cai Wu. 2013. "Effects of rice potassium level on the fecundity and expression of the vitellogenin gene of Nilaparvata lugens (Stål) (Hemiptera: Delphacidae)." *Journal of Asia-Pacific Entomology* 16 (4):411-414. doi: https://doi.org/10.1016/j.aspen.2013.06.001.

Lu, Zhong-Xian, Kong-Luen Heong, Xiao-Ping Yu, and Cui Hu. 2004. "Effects of Plant Nitrogen on Ecological Fitness of the Brown Planthopper, Nilaparvata lugens Stal. in Rice." *Journal of Asia-Pacific Entomology* 7 (1):97-104. doi: https://doi.org/10.1016/S1226-8615(08)60204-6.

Lundgren, Jonathan G., and Janet K. Fergen. 2011. "Enhancing predation of a subterranean insect pest: A conservation benefit of winter vegetation in agroecosystems." *Applied Soil Ecology* 51:9-16. doi: https://doi.org/10.1016/j.apsoil.2011.08.005.

Ma, Deying, Kevin Gorman, Greg Devine, Wanchun Luo, and Ian Denholm. 2007. "The biotype and insecticide-resistance status of whiteflies, Bemisia tabaci (Hemiptera: Aleyrodidae), invading cropping systems in Xinjiang Uygur Autonomous Region, northwestern China." *Crop Protection* 26 (4):612-617. doi: https://doi.org/10.1016/j.cropro.2006.04.027.

Ma, Fei, Zongze Ding, and Xianian Cheng. 2001. "Chaos and Predictable Time-Scale of the Brown Planthopper Nilaparvata Lugens (Stål) Occurrence System." *Journal of Asia-Pacific Entomology* 4 (1):67-74. doi: https://doi.org/10.1016/S1226-8615(08)60106-5.

Ma, Kangsheng, Fen Li, Pingzhuo Liang, Xuewei Chen, Ying Liu, Qiuling Tang, and Xiwu Gao. 2017. "RNA interference of Dicer-1 and Argonaute-1 increasing the sensitivity of Aphis gossypii Glover (Hemiptera: Aphididae) to plant allelochemical." *Pesticide Biochemistry and Physiology* 138:71-75. doi: https://doi.org/10.1016/j.pestbp.2017.03.003.

Ma, Ming-yong, Zhao-pu Peng, and Yuan He. 2012. "Effects of Temperature on Functional Response of Anagrus nilaparvatae Pang et Wang (Hymenoptera: Mymaridae) on the Eggs of Whitebacked Planthopper, Sogatella furcifera Horváth and Brown Planthopper, Nilaparvata lugens Stål." *Journal of Integrative Agriculture* 11 (8):1313-1320. doi: https://doi.org/10.1016/S2095-3119(12)60128-2.

Ma, Xiao-yan, Han-wen Wu, Wei-li Jiang, Ya-jie Ma, and Yan Ma. 2016. "Weed and insect control affected by mixing insecticides with glyphosate in cotton." *Journal of Integrative Agriculture* 15 (2):373-380. doi: https://doi.org/10.1016/S2095-3119(15)61188-1.

Mann, J. A., R. Harrington, N. Carter, and R. T. Plumb. 1997. "Control of aphids and barley yellow dwarf virus in spring-sown cereals." *Crop Protection* 16 (1):81-87. doi: https://doi.org/10.1016/S0261-2194(96)00068-3.

Mascarin, Gabriel Moura, Nilce Naomi Kobori, Eliane Dias Quintela, Steven Paul Arthurs, and Ítalo Delalibera Júnior. 2014. "Toxicity of non-ionic surfactants and interactions with fungal entomopathogens toward Bemisia tabaci biotype B." *BioControl* 59 (1):111-123. doi: 10.1007/s10526-013-9543-x.

Mascarin, Gabriel Moura, Nilce Naomi Kobori, Eliane Dias Quintela, and Italo Delalibera. 2013. "The virulence of entomopathogenic fungi against Bemisia tabaci biotype B (Hemiptera: Aleyrodidae) and their conidial production using solid substrate fermentation." *Biological Control* 66 (3):209-218. doi: https://doi.org/10.1016/j.biocontrol.2013.05.001.

Matsuura, Akira, and Masakazu Nakamura. 2014. "Development of neonicotinoid resistance in the cotton aphid Aphis gossypii (Hemiptera: Aphididae) in Japan." *Applied Entomology and Zoology* 49 (4):535-540. doi: 10.1007/s13355-014-0289-4.

Min, Sujeong, Si Woo Lee, Byeong-Ryeol Choi, Si Hyeock Lee, and Deok Ho Kwon. 2014. "Insecticide resistance monitoring and correlation analysis to select appropriate insecticides against Nilaparvata lugens (Stål), a migratory pest in Korea." *Journal of Asia-Pacific Entomology* 17 (4):711-716. doi: https://doi.org/10.1016/j.aspen.2014.07.005.

Morton, A., and F. Garcia-del-Pino. 2017a. "Laboratory and field evaluation of entomopathogenic nematodes for control of Agriotes obscurus (L.) (Coleoptera: Elateridae)." *Journal of Applied Entomology* 141 (4):241-246. doi: doi:10.1111/jen.12343.

Morton, A., and F. Garcia-del-Pino. 2017b. "Laboratory and field evaluation of entomopathogenic nematodes for control of Agriotes obscurus (L.) (Coleoptera: Elateridae)." *Journal of Applied Entomology* 141 (4):241-246. doi: doi:10.1111/jen.12343.

Mukanganyama, S., C. C. Figueroa, J. A. Hasler, and H. M. Niemeyer. 2003. "Effects of DIMBOA on detoxification enzymes of the aphid Rhopalosiphum padi (Homoptera: aphididae)." *J Insect Physiol* 49 (3):223-9.

Nanthakumar, M., V. Jhansi Lakshmi, V. Shashi Bhushan, S. M. Balachandran, and M. Mohan. 2012. "Decrease of rice plant resistance and induction of hormesis and carboxylesterase titre in brown planthopper, Nilaparvata lugens (Stål) by xenobiotics." *Pesticide Biochemistry and Physiology* 102 (2):146-152. doi: https://doi.org/10.1016/j.pestbp.2011.12.006.

Naranjo, Steven E. 2001. "Conservation and evaluation of natural enemies in IPM systems for Bemisia tabaci." *Crop Protection* 20 (9):835-852. doi: https://doi.org/10.1016/S0261-2194(01)00115-6.

Naveed, M., A. Salam, M. A. Saleem, and Ali H. Sayyed. 2008. "Effect of foliar applications of some insecticides onBemisia tabaci, predators and parasitoids: Implications in its management in Pakistan." *Phytoparasitica* 36 (4):377-387. doi: 10.1007/bf02980817.

Naveed, Muhammad, Abdus Salam, and Mushtaq Ahmad Saleem. 2007. "Contribution of cultivated crops, vegetables, weeds and ornamental plants in harboring of Bemisia tabaci (Homoptera: Aleyrodidae) and associated parasitoids (Hymenoptera: Aphelinidae) in cotton agroecosystem in Pakistan." *Journal of Pest Science* 80 (4):191-197. doi: 10.1007/s10340-007-0171-z.

Nielsen, Charlotte, and Tove Steenberg. 2004. "Entomophthoralean fungi infecting the bird cherry-oat aphid, Rhopalosiphum padi, feeding on its winter host bird cherry, Prunus padus." *Journal of Invertebrate Pathology* 87 (1):70-73. doi: https://doi.org/10.1016/j.jip.2004.05.003.

Opatovsky, Itai, Eric Chapman, Phyllis Weintraub, Yael Lubin, and James Harwood. 2012. *Molecular characterization of the differential role of immigrant and agrobiont generalist predators in pest suppression*. Vol. 63.

Parker, William E. 1994. "Evaluation of the use of food baits for detecting wireworms (Agriotes spp., Coleoptera: Elateridae) in fields intended for arable crop production." *Crop Protection* 13 (4):271-276. doi: https://doi.org/10.1016/0261-2194(94)90014-0.

Parker, William E. 1996. "The development of baiting techniques to detect wireworms (Agriotes spp., Coleoptera: Elateridae) in the field, and the relationship between bait-trap catches and wireworm damage to potato." *Crop Protection* 15 (6):521-527. doi: https://doi.org/10.1016/0261-2194(96)00020-8.

Parry, Hazel R., Andrew J. Evans, and Derek Morgan. 2006. "Aphid population response to agricultural landscape change: A spatially explicit, individual-based model." *Ecological Modelling* 199 (4):451-463. doi: https://doi.org/10.1016/j.ecolmodel.2006.01.006.

Pilz, Christina, Siegfried Keller, Ulrich Kuhlmann, and Stefan Toepfer. 2009. "Comparative efficacy assessment of fungi, nematodes and insecticides to control western corn rootworm larvae in maize." *BioControl* 54 (5):671-684. doi: 10.1007/s10526-009-9209-x.

Piyaratne, M. K. D. K., Huiyan Zhao, and Qingxiang Meng. 2013. "APHIDSim: A population dynamics model for wheat aphids based on swallowtail catastrophe theory." *Ecological Modelling* 253:9-16. doi: https://doi.org/10.1016/j.ecolmodel.2012.12.032.

Pluschkell, U., A. R. Horowitz, and I. Ishaaya. 1999. "Effect of milbemectin on the sweetpotato whitefly,Bemisia tabad." *Phytoparasitica* 27 (3):183. doi: 10.1007/bf02981457.

Puinean, Alin M., Ian Denholm, Neil S. Millar, Ralf Nauen, and Martin S. Williamson. 2010. "Characterisation of imidacloprid resistance mechanisms in the brown planthopper, Nilaparvata lugens Stål (Hemiptera: Delphacidae)." *Pesticide Biochemistry and Physiology* 97 (2):129-132. doi: https://doi.org/10.1016/j.pestbp.2009.06.008.

Rahatkhah, Zahra, Javad Karimi, Mohammad Ghadamyari, and Maurizio F. Brivio. 2015. "Immune defenses of Agriotes lineatus larvae against entomopathogenic nematodes." *BioControl* 60 (5):641-653. doi: 10.1007/s10526-015-9678-z.

Rashid, Md Mamunur, Mahbuba Jahan, and Khandakar Shariful Islam. 2016. "Impact of Nitrogen, Phosphorus and Potassium on Brown Planthopper and Tolerance of Its Host Rice Plants." *Rice Science* 23 (3):119-131. doi: https://doi.org/10.1016/j.rsci.2016.04.001.

Reddy, Gadi V. P., Khanobporn Tangtrakulwanich, Shaohui Wu, John H. Miller, Victoria L. Ophus, Julie Prewett, and Stefan T. Jaronski. 2014. "Evaluation of the effectiveness of entomopathogens for the management of wireworms (Coleoptera: Elateridae) on spring wheat." *Journal of Invertebrate Pathology* 120:43-49. doi: https://doi.org/10.1016/j.jip.2014.05.005.

Ritter, Claudia, and Ellen Richter. 2013. "Control methods and monitoring of Agriotes wireworms (Coleoptera: Elateridae)." *Journal of Plant Diseases and Protection* 120 (1):4-15. doi: 10.1007/bf03356448.

Ritter, Claudia, Ellen Richter, Irena Knölck, and Kai-Uwe Katroschan. 2014. "Laboratory studies on the effect of calcium cyanamide on wireworms (Agriotes ustulatus, Coleoptera: Elateridae)." *Journal of Plant Diseases and Protection* 121 (3):133-137. doi: 10.1007/bf03356501.

Roh, Hyun Sik, Junheon Kim, Eun-Sik Shin, Dong Woon Lee, Ho Yul Choo, and Chung Gyoo Park. 2015a. "Bioactivity of sandalwood oil (Santalum austrocaledonicum) and its main components against the cotton aphid, Aphis gossypii." *Journal of Pest Science* 88 (3):621-627. doi: 10.1007/s10340-014-0631-1.

Roh, Hyun Sik, Junheon Kim, Eun-Sik Shin, Dong Woon Lee, Ho Yul Choo, and Chung Gyoo Park. 2015b. "Bioactivity of sandalwood oil (Santalum austrocaledonicum) and its main components against the cotton aphid, Aphis gossypii." *Journal of Pest Science* 88 (3):621-627. doi: 10.1007/s10340-014-0631-1.

Roschewitz, Indra, Melanie Hücker, Teja Tscharntke, and Carsten Thies. 2005. "The influence of landscape context and farming practices on parasitism of cereal aphids." *Agriculture, Ecosystems & Environment* 108 (3):218-227. doi: https://doi.org/10.1016/j.agee.2005.02.005.

Rossing, W. A. H., R. A. Daamen, and E. M. T. Hendrix. 1994. "Framework to support decisions on chemical pest control under uncertainty, applied to aphids and brown rust in winter wheat." *Crop Protection* 13 (1):25-34. doi: https://doi.org/10.1016/0261-2194(94)90132-5.

Rusch, Adrien, Rebecca Chaplin-Kramer, Mary M. Gardiner, Violetta Hawro, John Holland, Douglas Landis, Carsten Thies, Teja Tscharntke, Wolfgang W. Weisser, Camilla Winqvist, Megan Woltz, and Riccardo Bommarco. 2016. "Agricultural landscape simplification reduces natural pest control: A quantitative synthesis." *Agriculture, Ecosystems & Environment* 221:198-204. doi: https://doi.org/10.1016/j.agee.2016.01.039.

Sampson, Kimberly, Jelena Zaitseva, Maria Stauffer, Brian Vande Berg, Rong Guo, Daniel Tomso, Brian McNulty, Nalini Desai, and Deepa Balasubramanian. 2017. "Discovery of a novel insecticidal protein from Chromobacterium piscinae, with activity against Western Corn Rootworm, Diabrotica virgifera virgifera." *Journal of Invertebrate Pathology* 142:34-43. doi: https://doi.org/10.1016/j.jip.2016.10.004.

Saussure, Stéphanie, Manuel Plantegenest, Jean-Baptiste Thibord, Philippe Larroudé, and Sylvain Poggi. 2015. "Management of wireworm damage in maize fields using new, landscape-scale strategies." *Agronomy for Sustainable Development* 35 (2):793-802. doi: 10.1007/s13593-014-0279-5.

Schallhart, Nikolaus, Manuel Josef Tusch, Corinna Wallinger, Karin Staudacher, and Michael Traugott. 2012. "Effects of plant identity and diversity on the dietary choice of a soil‐living insect herbivore." *Ecology* 93 (12):2650-2657. doi: doi:10.1890/11-2067.1.

Schumann, M., A.Patel, and S. Vidal. 2013. "Evaluation of an attract and kill strategy for western corn rootworm larvae." *Applied Soil Ecology* 64:178-189. doi: https://doi.org/10.1016/j.apsoil.2012.12.007.

Schumann, M., S. Toepfer, M. Vemmer, A. Patel, U. Kuhlmann, and S. Vidal. 2014. "Field evaluation of an attract and kill strategy against western corn rootworm larvae." *Journal of Pest Science* 87 (2):259-271. doi: 10.1007/s10340-013-0551-5.

Schütz, Kirsten, Michael Bonkowski, and Stefan Scheu. 2008. "Effects of Collembola and fertilizers on plant performance (Triticum aestivum) and aphid reproduction (Rhopalosiphum padi)." *Basic and Applied Ecology* 9 (2):182-188. doi: https://doi.org/10.1016/j.baae.2006.07.003.

Senthil-Nathan, Sengottayan, Man-Young Choi, Chae-Hoon Paik, Hong-Yul Seo, and Kandaswamy Kalaivani. 2009. "Toxicity and physiological effects of neem pesticides applied to rice on the Nilaparvata lugens Stål, the brown planthopper." *Ecotoxicology and Environmental Safety* 72 (6):1707-1713. doi: https://doi.org/10.1016/j.ecoenv.2009.04.024.

Senthil-Nathan, Sengottayan, Kandaswamy Kalaivani, Man-Young Choi, and Chae-Hoon Paik. 2009. "Effects of jasmonic acid-induced resistance in rice on the plant brownhopper, Nilaparvata lugens Stål (Homoptera: Delphacidae)." *Pesticide Biochemistry and Physiology* 95 (2):77-84. doi: https://doi.org/10.1016/j.pestbp.2009.07.001.

Sequeira, Richard V., and Steven E. Naranjo. 2008. "Sampling and management of Bemisia tabaci (Genn.) biotype B in Australian cotton." *Crop Protection* 27 (9):1262-1268. doi: https://doi.org/10.1016/j.cropro.2008.04.002.

Shentu, Xu-Ping, Dan-Ting Li, Jian-Feng Xu, Liang She, and Xiao-Ping Yu. 2016. "Effects of fungicides on the yeast-like symbiotes and their host, Nilaparvata lugens Stål (Hemiptera: Delphacidae)." *Pesticide Biochemistry and Physiology* 128:16-21. doi: https://doi.org/10.1016/j.pestbp.2015.10.010.

Silvie, Pierre Jean, Alain Renou, Samuel Vodounnon, Gustave Bonni, Moïse Obayomi Adegnika, Omer Héma, Patrick Prudent, Julie Sorèze, Germain Ochou Ochou, Mamoutou Togola, Djibril Badiane, Abdoulaye Ndour, Pikassalé Komlan Akantetou, Bassarou Ayeva, and Thierry Brévault. 2013. "Threshold-based interventions for cotton pest control in West Africa: What's up 10 years later?" *Crop Protection* 43:157-165. doi: https://doi.org/10.1016/j.cropro.2012.09.006.

Sohrabi, Fariba, Parviz Shishehbor, Moosa Saber, and Mohammad Said Mosaddegh. 2012. "Lethal and sublethal effects of buprofezin and imidacloprid on the whitefly parasitoid Encarsia inaron (Hymenoptera: Aphelinidae)." *Crop Protection* 32:83-89. doi: https://doi.org/10.1016/j.cropro.2011.10.005.

Spangenberg, J. H., J. M. Douguet, J. Settele, and K. L. Heong. 2015. "Escaping the lock-in of continuous insecticide spraying in rice: Developing an integrated ecological and socio-political DPSIR analysis." *Ecological Modelling* 295:188-195. doi: https://doi.org/10.1016/j.ecolmodel.2014.05.010.

Staudacher, Karin, Nikolaus Schallhart, Peter Pitterl, Corinna Wallinger, Nina Brunner, Marion Landl, Bernhard Kromp, Johann Glauninger, and Michael Traugott. 2013. "Occurrence of Agriotes wireworms in Austrian agricultural land." *Journal of Pest Science* 86 (1):33-39. doi: 10.1007/s10340-011-0393-y.

Steinkraus, Donald C., and Gabriele O. Boys. 2005. "Mass harvesting of the entomopathogenic fungus, Neozygites fresenii, from natural field epizootics in the cotton aphid, Aphis gossypii." *Journal of Invertebrate Pathology* 88 (3):212-217. doi: https://doi.org/10.1016/j.jip.2005.01.008.

Stenberg, J. A., M. Heil, I. Ahman, and C. Bjorkman. 2015. "Optimizing Crops for Biocontrol of Pests and Disease." *Trends Plant Sci* 20 (11):698-712. doi: 10.1016/j.tplants.2015.08.007.

Sufyan, Muhammad, Daniel Neuhoff, and Lorenzo Furlan. 2011. "Assessment of the range of attraction of pheromone traps to Agriotes lineatus and Agriotes obscurus." *Agricultural and Forest Entomology* 13 (3):313-319. doi: doi:10.1111/j.1461-9563.2011.00529.x.

Sy Mohamad, Sharifah Fathiyah, Shahril Mohamad, and Azila Abdul Aziz. 2013. "The Susceptibility of Aphids, Aphis gossypii Glover to Lauric Acid based Natural Pesticide." *Procedia Engineering* 53:20-28. doi: https://doi.org/10.1016/j.proeng.2013.02.004.

Szalai, Márk, József Kiss, Szilvia Kövér, and Stefan Toepfer. 2014. "Simulating crop rotation strategies with a spatiotemporal lattice model to improve legislation for the management of the maize pest Diabrotica virgifera virgifera." *Agricultural Systems* 124:39-50. doi: https://doi.org/10.1016/j.agsy.2013.10.009.

Tan, Ye, Mufei Zhu, Wenyan Xu, Wenwu Zhou, Dongdong Lu, Hanwu Shang, and Zengrong Zhu. 2017. "Influence of water-stressed rice on feeding behavior of brown planthopper, Nilaparvata lugens (Stål)." *Journal of Asia-Pacific Entomology* 20 (2):665-670. doi: https://doi.org/10.1016/j.aspen.2017.03.012.

Tang, Bin, Mengmeng Yang, Qida Shen, Yanxia Xu, Huijuan Wang, and Shigui Wang. 2017. "Suppressing the activity of trehalase with validamycin disrupts the trehalose and chitin biosynthesis pathways in the rice brown planthopper, Nilaparvata lugens." *Pesticide Biochemistry and Physiology* 137:81-90. doi: https://doi.org/10.1016/j.pestbp.2016.10.003.

Toepfer, Stefan, Benedikt Kurtz, and Ulrich Kuhlmann. 2010. "Influence of soil on the efficacy of entomopathogenic nematodes in reducing Diabrotica virgifera virgifera in maize." *Journal of Pest Science* 83 (3):257-264. doi: 10.1007/s10340-010-0293-6.

Trisyono, Y. Andi, Valentina E. F. Aryuwandari, Teguh Rahayu, and Edhi Martono. 2017. "Effects of etofenprox applied at the sublethal concentration on the fecundity of rice brown planthopper, Nilaparvata lugens." *Journal of Asia-Pacific Entomology* 20 (2):547-551. doi: https://doi.org/10.1016/j.aspen.2017.03.013.

Tóth, Miklós. 2013. "Pheromones and attractants of click beetles: an overview." *Journal of Pest Science* 86 (1):3-17. doi: 10.1007/s10340-012-0429-y.

Tóth, Miklós, Lorenzo Furlan, József Vuts, István Szarukán, István Ujváry, Venyamin G. Yatsynin, Till Tolasch, and Wittko Francke. 2015. "Geranyl hexanoate, the female-produced pheromone of Agriotes sordidus Illiger (Coleoptera: Elateridae) and its activity on both sexes." *Chemoecology* 25 (1):1-10. doi: 10.1007/s00049-014-0170-5.

Tóth, Miklós, Lorenzo Furlan, Amália Xavier, József Vuts, Teodora Toshova, Mitko Subchev, István Szarukán, and Venyamin Yatsynin. 2007. "New Sex Attractant Composition for the Click Beetle Agriotes proximus: Similarity to the Pheromone of Agriotes lineatus." *Journal of Chemical Ecology* 34 (1):107. doi: 10.1007/s10886-007-9398-7.

Tóth, Miklós, Lorenzo Furlan, Venyamin G. Yatsynin, István Ujváry, István Szarukán, Zoltán Imrei, Mitko Subchev, Till Tolasch, and Wittko Francke. 2002. "Identification of Sex Pheromone Composition of Click Beetle Agriotes brevis Candeze." *Journal of Chemical Ecology* 28 (8):1641-1652. doi: 10.1023/a:1019984714858.

van Herk, Willem G., and Robert S. Vernon. 2013. "Wireworm damage to wheat seedlings: effect of temperature and wireworm state." *Journal of Pest Science* 86 (1):63-75. doi: 10.1007/s10340-012-0461-y.

Vasileiadis, V. P., M. Sattin, S. Otto, A. Veres, Z. Pálinkás, R. Ban, X. Pons, P. Kudsk, R. van der Weide, E. Czembor, A. C. Moonen, and J. Kiss. 2011. "Crop protection in European maize-based cropping systems: Current practices and recommendations for innovative Integrated Pest Management." *Agricultural Systems* 104 (7):533-540. doi: https://doi.org/10.1016/j.agsy.2011.04.002.

Veres, Andrea, Sandrine Petit, Cyrille Conord, and Claire Lavigne. 2013. "Does landscape composition affect pest abundance and their control by natural enemies? A review." *Agriculture, Ecosystems & Environment* 166:110-117. doi: https://doi.org/10.1016/j.agee.2011.05.027.

Vernon, Robert S., Roderick P. Blackshaw, Willem G. van Herk, and Markus Clodius. 2014. "Mass trapping wild Agriotes obscurus and Agriotes lineatus males with pheromone traps in a permanent grassland population reservoir." *Agricultural and Forest Entomology* 16 (3):227-239. doi: doi:10.1111/afe.12058.

Vernon, Robert S., and Willem G. van Herk. 2013. "Physical exclusion of adult click beetles from wheat with an exclusion trench." *Journal of Pest Science* 86 (1):77-83. doi: 10.1007/s10340-012-0472-8.

Vernon, Robert S., Willem G. van Herk, Markus Clodius, and Jeff Tolman. 2016. "Companion planting attract-and-kill method for wireworm management in potatoes." *Journal of Pest Science* 89 (2):375-389. doi: 10.1007/s10340-015-0707-6.

Viscarret, Mariana M., and Silvia N. López. 2004. "Biological studies on Encarsia porteri (Mercet) (Hymenoptera: Aphelinidae) an heterotrophic parasitoid of the Bemisia tabaci (Gennadius) (Hemiptera: Aleyrodidae) complex." *Biological Control* 30 (2):236-240. doi: https://doi.org/10.1016/j.biocontrol.2003.10.002.

Vu, Van Hanh, Suk Il Hong, and Keun Kim. 2007. "Selection of Entomopathogenic Fungi for Aphid Control." *Journal of Bioscience and Bioengineering* 104 (6):498-505. doi: https://doi.org/10.1263/jbb.104.498.

Vuts, József, Lorenzo Furlan, Éva Bálintné Csonka, Christine M Woodcock, John C Caulfield, Patrick Mayon, John A Pickett, Michael A Birkett, and Miklós Tóth. 2014. "Development of a female attractant for the click beetle pest Agriotes brevis." *Pest Management Science* 70 (4):610-614. doi: doi:10.1002/ps.3589.

Vuts, József, Till Tolasch, Lorenzo Furlan, Éva Bálintné Csonka, Tamás Felföldi, Károly Márialigeti, Teodora B. Toshova, Mitko Subchev, Amália Xavier, and Miklós Tóth. 2012. "Agriotes proximus and A. lineatus (Coleoptera: Elateridae): a comparative study on the pheromone composition and cytochrome c oxidase subunit I gene sequence." *Chemoecology* 22 (1):23-28. doi: 10.1007/s00049-011-0091-5.

Wang, Wanlei, Yong Liu, Julian Chen, Xianglong Ji, Haibo Zhou, and Guang Wang. 2009. "Impact of intercropping aphid-resistant wheat cultivars withoilseed rape on wheat aphid (Sitobion avenae) and its natural enemies." *Acta Ecologica Sinica* 29 (3):186-191. doi: https://doi.org/10.1016/j.chnaes.2009.07.009.

Westphal, Catrin, Stefan Vidal, Finbarr G. Horgan, Geoff M. Gurr, Monina Escalada, Ho Van Chien, Teja Tscharntke, Kong Luen Heong, and Josef Settele. 2015. "Promoting multiple ecosystem services with flower strips and participatory approaches in rice production landscapes." *Basic and Applied Ecology* 16 (8):681-689. doi: https://doi.org/10.1016/j.baae.2015.10.004.

Wu, Wenqi, M. K. D. K. Piyaratne, Huiyan Zhao, Chunlong Li, Zuqing Hu, and Xiangshun Hu. 2014. "Butterfly catastrophe model for wheat aphid population dynamics: Construction, analysis and application." *Ecological Modelling* 288:55-61. doi: https://doi.org/10.1016/j.ecolmodel.2014.05.017.

Xian, Xiaoqing, Baoping Zhai, Xiaoxi Zhang, Xianian Cheng, and Jianqiang Wang. 2007. "Teleconnection between the early immigration of brown planthopper (Nilaparvata lugens Stål) and ENSO indices: implication for its medium- and long-term forecast." *Acta Ecologica Sinica* 27 (8):3144-3154. doi: https://doi.org/10.1016/S1872-2032(07)60069-9.

Xie, Yongshou, J. Thor Arnason, Bernard J. R. Philogéne, Jeffrey Atkinson, and Peter Morand. 1992. "Behavioral responses of western corn rootworm larvae to naturally occurring and synthetic hydroxamic acids." *Journal of Chemical Ecology* 18 (7):945-957. doi: 10.1007/bf00980055.

Xu, Hong-Xing, Xu-Song Zheng, Ya-Jun Yang, Jun-Ce Tian, Yan-Hui Lu, Keng-Hong Tan, Kong-Luen Heong, and Zhong-Xian Lu. 2015. "Methyl eugenol bioactivities as a new potential botanical insecticide against major insect pests and their natural enemies on rice (Oriza sativa)." *Crop Protection* 72:144-149. doi: https://doi.org/10.1016/j.cropro.2015.03.017.

Xu, Lu, Chun-Qing Zhao, De-Jin Xu, Guang-Chun Xu, Xiao-Long Xu, Zhao-Jun Han, Ya-Nan Zhang, and Zhong-Yan Gu. 2017. "RNAi suppression of nuclear receptor genes results in increased susceptibility to sulfoxaflor in brown planthopper, Nilaparvata lugens." *Journal of Asia-Pacific Entomology* 20 (2):645-653. doi: https://doi.org/10.1016/j.aspen.2017.03.022.

Xu, Man-yu, Ting Zhou, Yan-ying Zhao, Jia-bao Li, Heng Xu, Han-song Dong, and Chun-ling Zhang. 2014. "Transgenic Expression of a Functional Fragment of Harpin Protein Hpa1 in Wheat Represses English Grain Aphid Infestation." *Journal of Integrative Agriculture* 13 (12):2565-2576. doi: https://doi.org/10.1016/S2095-3119(13)60735-2.

Yang, Nian-Wan, Ai-Lian Li, Fang-Hao Wan, Wan-Xue Liu, and Dan Johnson. 2010. "Effects of plant essential oils on immature and adult sweetpotato whitefly, Bemisia tabaci biotype B." *Crop Protection* 29 (10):1200-1207. doi: https://doi.org/10.1016/j.cropro.2010.05.006.

Yang, Nian-Wan, and Fang-Hao Wan. 2011. "Host suitability of different instars of Bemisia tabaci biotype B for the parasitoid Eretmocerus hayati." *Biological Control* 59 (2):313-317. doi: https://doi.org/10.1016/j.biocontrol.2011.07.019.

Yang, Ya-jun, Bi-qin Dong, Hong-xing Xu, Xu-song Zheng, K. L. Heong, and Zhong-xian Lu. 2014. "Susceptibility to Insecticides and Ecological Fitness in Resistant Rice Varieties of Field Nilaparvata lugens Stål Population Free from Insecticides in Laboratory." *Rice Science* 21 (3):181-186. doi: https://doi.org/10.1016/S1672-6308(13)60181-X.

Yao, Feng-Luan, Min-Sheng You, Liette Vasseur, Guang Yang, and Yun-Kai Zheng. 2012. "Polycultural manipulation for better regulation of planthopper populations in irrigated rice-based ecosystems." *Crop Protection* 34:104-111. doi: https://doi.org/10.1016/j.cropro.2011.12.003.

Yao, Feng-Luan, Yu Zheng, Jian-Wei Zhao, Nicolas Desneux, Yu-Xian He, and Qi-Yong Weng. 2015. "Lethal and sublethal effects of thiamethoxam on the whitefly predator Serangium japonicum (Coleoptera: Coccinellidae) through different exposure routes." *Chemosphere* 128:49-55. doi: https://doi.org/10.1016/j.chemosphere.2015.01.010.

Zhang, Meng, Xianfeng Qiao, Xiong Peng, and Maohua Chen. 2016. "Variation of resistance and susceptibility in wheat cultivars to different populations of Rhopalosiphum padi (Hemiptera: Aphididae) in China." *Journal of Asia-Pacific Entomology* 19 (2):307-311. doi: https://doi.org/10.1016/j.aspen.2016.03.006.

Zhang, Xiao-ming, Nian-wan Yang, Fang-hao Wan, and Gabor L. Lövei. 2014. "Density and Seasonal Dynamics of Bemisia tabaci (Gennadius) Mediterranean on Common Crops and Weeds Around Cotton Fields in Northern China." *Journal of Integrative Agriculture* 13 (10):2211-2220. doi: https://doi.org/10.1016/S2095-3119(13)60613-9.

Zhang, Xiaolei, Xun Liao, Kaikai Mao, Kaixiong Zhang, Hu Wan, and Jianhong Li. 2016. "Insecticide resistance monitoring and correlation analysis of insecticides in field populations of the brown planthopper Nilaparvata lugens (stål) in China 2012–2014." *Pesticide Biochemistry and Physiology* 132:13-20. doi: https://doi.org/10.1016/j.pestbp.2015.10.003.

Zheng, Yue-Lian, Lu Xu, Jin-Cai Wu, Jing-Lan Liu, and He-Lin DuanMu. 2007. "Time of occurrence of hopperburn symptom on rice following root and leaf cutting and fertilizer application with brown planthopper, Nilaparvata lugens (stål) infestation." *Crop Protection* 26 (2):66-72. doi: https://doi.org/10.1016/j.cropro.2006.04.001.

Zhou, Hai-bo, Ju-lian Chen, Yong Liu, Frédéric Francis, Eric Haubruge, Claude Bragard, Jing-rui Sun, and Deng-fa Cheng. 2013. "Influence of Garlic Intercropping or Active Emitted Volatiles in Releasers on Aphid and Related Beneficial in Wheat Fields in China." *Journal of Integrative Agriculture* 12 (3):467-473. doi: https://doi.org/10.1016/S2095-3119(13)60247-6.

Zhou, Ke, Jikun Huang, Xiangzheng Deng, Wopke van der Werf, Wei Zhang, Yanhui Lu, Kongming Wu, and Feng Wu. 2014. "Effects of land use and insecticides on natural enemies of aphids in cotton: First evidence from smallholder agriculture in the North China Plain." *Agriculture, Ecosystems & Environment* 183:176-184. doi: https://doi.org/10.1016/j.agee.2013.11.008.

Östman, Örjan, Barbara Ekbom, and Janne Bengtsson. 2001. "Landscape heterogeneity and farming practice influence biological control." *Basic and Applied Ecology* 2 (4):365-371. doi: https://doi.org/10.1078/1439-1791-00072.
